# Multilocus Molecular Phylogeny of the *Umbilicaria aprina* Group (Umbilicariaceae, Lichenized Ascomycota) Supports Species Level and Neo-Endemic Status of *Umbilicaria krascheninnikovii*

**DOI:** 10.3390/plants13050729

**Published:** 2024-03-04

**Authors:** Evgeny A. Davydov, Dmitry E. Himelbrant, Ekaterina S. Kuznetsova, Irina S. Stepanchikova, Lidia S. Yakovchenko

**Affiliations:** 1 Altai State University, Lenin Ave. 61, Barnaul 656049, Russia; 2 Komarov Botanical Institute, Professor Popov St. 2, St. Petersburg 197376, Russia; d_brant@mail.ru (D.E.H.); igel_kuzn@mail.ru (E.S.K.); stepa_ir@mail.ru (I.S.S.); 3 Saint-Petersburg State University, Universitetskaya Emb. 7/9, St. Petersburg 199034, Russia; 4 Federal Scientific Center of the East Asia Terrestrial Biodiversity FEB RAS, 100th Anniversary of Vladivostok Avenue, 159, Vladivostok 690022, Russia; lidiyakovchenko@mail.ru

**Keywords:** molecular phylogeny, morphology, thalloconidia, *Umbilicaria polaris*

## Abstract

The Northeast Asian endemic species of lichen-forming fungus *Umbilicaria krascheninnikovii* is herein discussed in the global context of biogeography and phylogeny of the *U. aprina* group. The name *U. krascheninnikovii* has been erroneously used by lichenologists for *Umbilicaria* spp. from high latitudes or altitudes worldwide, as there are omphalodisc apothecia and rough “crystals” of a necral layer on the upper surface. To test the monophyly and phylogenetic relationships within the *U. aprina* group, four independent DNA regions (*nrITS*/*5.8S*, *RPB2*, *mtLSU*, and *mtSSU*) were used for six rare species, including a dozen specimens of *U. krascheninnikovii* from its locus classicus in Kamchatka. The study is based on the phylograms obtained using maximum likelihood and a Bayesian phylogenetic inference framework. As a result of phylogenetic and biogeographic analyses, it was shown that *U. krascheninnikovii* is a neo-endemic of the areas of modern volcanism in Kamchatka, Japan, as well as in the Kurile Islands, where this species was recorded for the first time. The morphology of *U. krascheninnikovii* is herein described and illustrated. Increasing the role of the sexual process and reducing asexual thalloconidiogenesis are shown to be apomorphic traits in the *U. aprina* group. The combination of sexual and asexual reproduction provides adaptive advantages in changing environmental conditions.

## 1. Introduction

Lichens are symbiotic organisms composed of a fungal partner, the mycobiont, and one or more photosynthetic partners, i.e., the photobionts [1]. Lichens show distinctive patterns of distribution, similar to other major groups of organisms. Generally, the distribution patterns of lichens are wider than those of vascular plants, e.g., [2,3]. Nevertheless, some lichens appear to have very restricted ranges [4]. An endemic element is often present in the lichen mycobiota of a particular geographical region. High endemism is characterized for relatively isolated territories such as islands, peninsulas, isolated mountain massifs, etc. A molecular–phylogenetic approach is changing our view on the biogeographic patterns of lichens, revealing diverse lineages of cryptic or overlooked species within some species previously considered to be widespread [5,6,7,8,9,10] and demonstrating that high diversity and endemism in insular lichens are common phenomena. Regional endemism is known, e.g., for Papua New Guinea [11], Macaronesia [12,13], Galapagos [14,15], Hawaii [16], Socotra [17], the Caribbean [18], Madagascar [19], Japan [20,21], New Zealand [22], and Tasmania [23]. The mentioned studies show that the level of island endemism in some lichen genera can reach up to 100%. The origin of endemism in lichens has historical, ecological, and biological reasons, such as the age of formation and colonization of islands or mountains [24,25], isolation of the territory, specificity of environmental conditions [16,26], and the ability of propagules to long-distance dispersal [27,28,29].

Species of Umbilicariaceae Chevall. are predominantly saxicolous lichens, mostly found in regions of higher latitudes or altitudes worldwide. Multilocus phylogenies resulted in a new generic concept of the family, which currently includes three genera, namely *Fulgidea* Bendiksby & Timdal, *Umbilicaria* Hoffm., and *Xylopsora* Bendiksby & Timdal, comprising together about one hundred species of mostly umbilicate growth habits [30,31]. According to a molecular clock analysis [32], Umbilicariales branched off from the main lineages of Lecanoromycetes prior to the major diversification of the latter ca. 191 million years ago. The worldwide distribution of centers of species diversity and endemism of *Umbilicaria* also suggests their old phylogenetic age. Umbilicariaceae includes species with mostly Holarctic or Bipolar distribution with endemic elements and in Europe, East Asia, North and South America, New Zealand, and Antarctica, i.e., on every continent [26,31,33,34,35,36,37,38,39,40,41].

*Umbilicaria krascheninnikovii* (Savicz) Zahlbr. is an enigmatic species endemic to East Asia. The holotype material of *U. krascheninnikovii* is represented by very small and mostly juvenile thalli, and there have been just single collections since the original description of the species by Savicz [42] until the comprehensive investigation of the lichens of Kamchatka by Himelbrant et al. [43,44,45,46]. *Gyrophora krascheninnikovii* Savicz (≡*Umbilicaria krascheninnikovii*) was treated by Scholander [47] as a priority name for *G. hultenii* DuRietz [48] and *Gyrophora polaris* Schol. [49]. Llano [35], following Scholander’s opinion on *Umbilicaria* (*Omphalodiscus*) *krascheninnikovii*, carefully compared the descriptions of the mentioned species in his monograph and came to the same conclusions. The name *U. krascheninnikovii* was erroneously used by lichenologists for *Umbilicaria* spp. from high latitudes and high latitudes worldwide (e.g., [35,50,51,52,53,54,55,56]). The mentioned authors treated the species as being closely related to *U. decussata* (Vill.) Zahlbr. but clearly separated from the latter species by its pale lower surface and abundance of apothecia. However, the holotype of *Gyrophora krascheninnikovii* was not examined by the mentioned authors. Contrary to this, Wei and Jiang [36], based on the holotype examination, treated *U. krascheninnikovii* as a separate species distributed in Kamchatka and Japan. Davydov et al. [31] included material of the putative true *U. krascheninnikovii* from Kamchatka to a phylogenetic study and showed that it belongs to the “*U. aprina*” group, which combines several closely related species, i.e., *U. africana* (Jatta) Krog et Swinscow, *U. antarctica* Frey et Lamb, *U. aprina* Nyl., *U. formosana* Frey, *U. kappeni* Sancho et al., and *U. rhizinata* (Frey et Poelt) Krzewicka within *Umbilicaria* subg. *Umbilicaria*. This species complex is quite poorly investigated and is characterized by polar–high mountain distribution and different modes of propagation. Species of the *U. aprina* group develop rather characteristic mitosporic dispersal units, so-called “thalloconidia”, which can be non-septate or septate and were shown to be highly species-specific [57,58].

*Umbilicaria krascheninnikovii* was described from Kamchatka [42]. The Kamchatka Peninsula, with 30 active volcanoes, is one of the most volcanically active regions in the world. Modern volcanism has a great impact on the environment due to thermal activity and eruptions. Since the beginning of the 20th century, more than 180 eruptions of 15 volcanoes have occurred in Kamchatka. The productivity of the Klyuchevskoy volcano averages 60 million tons per year [59]. It is noteworthy that the Kamchatka Peninsula is connected to the mainland by a relatively narrow bridge in the north, and therefore, it is quite isolated from the rest of the continent, almost like an island. The Kamchatka shores are washed by the Pacific Ocean and Sea of Okhotsk and have an oceanic climate. Due to the presence of the meridian-oriented Sredinny and Eastern Volcanic Ridges, the climate of the inner part of the peninsula is more continental. Besides 29 active and about 300 extinct volcanoes, Kamchatka also has 446 glaciers covering about 900 km^2^ (the total area of the peninsula is 350,000 km^2^). Both volcanoes and glaciers influence the climate [60].

Endemic taxa are of considerable biogeographical interest; they may either represent the emergence of “new” species from ancestors over a period of isolation, i.e., “neo-endemics”, or reflect an “old” or “relict” distribution of a group surviving after widespread extinctions, i.e., “paleo-endemics” [3,61,62]. Phenotypic investigations may help to interpret the status and biological background of endemism, but due to the wide-ranging homoplasy of phenotypic traits in lichens, the value of such evidence is limited. Molecular genetic data, particularly DNA sequence data from several unlinked loci, and phylogenetics are increasingly being used to reveal the evolution of species and their complexes (e.g., [14,16,26,63]).

The present study proves the species status of *Umbilicaria krascheninnikovii* based on a multilocus phylogeny of the *U. aprina* group and improves our understanding of the species circumscription and its phylogenetic relationships using a combination of morphological and DNA sequence data.

## 2. Results

### 2.1. The Phylogenetic Study

**Figure 1:**
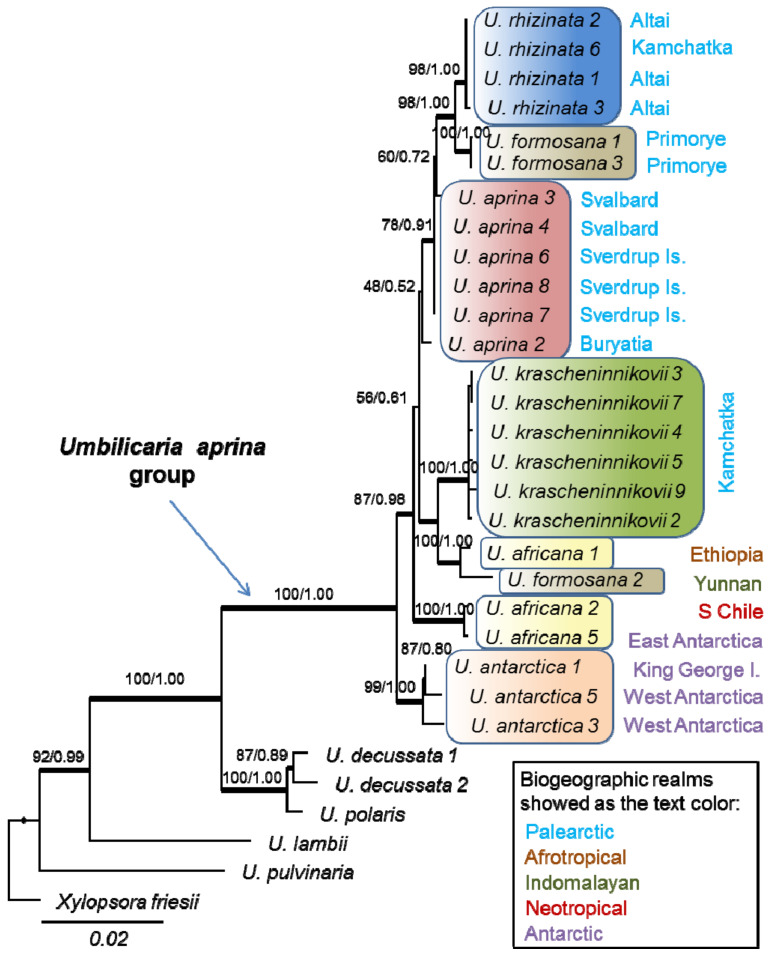
Reconstruction of *ITS*+*mtLSU*+*mtSSU*+*RPB2* phylogeny of the *Umbilicaria aprina* group based on maximum likelihood analysis with IQ-TREE algorithm. GenBank accession numbers are given in Table S1. The number in each node represents bootstrap support (BS) and posterior probability (PP). Branch lengths represent the estimated number of substitutions per site assuming the respective models of substitution. An exception is the branch with a black dot, which was shortened to reduce the overall figure size.

For the phylogenetic analyses, we used 132 original sequences, 73 of which were obtained during this study and 59 obtained in earlier investigations [31] and deposited in GenBank (Table S1). The major taxon grouping was similar in the phylogenetic reconstructions of all markers (Figures S1–S4) despite the fact that the different datasets do not include the same specimens for all species (Table S1). The *ITS* phylogram (Figure S1) includes almost all specimens and is highly resolved, i.e., contains several well-supported lineages for separate species or their regional populations, but the relationships in the backbone are not statistically supported. The phylogram based on the more conservative *mtLSU* and *mtSSU* markers segregated identical or slightly variable sequences at the species level (Figures S2 and S3), but some taxa are unresolved. The *RPB2* phylogram (Figure S4) had a well-supported backbone but includes just a small fraction of specimens, 1–2 for every species. A concatenated *ITS*+*RPB2*+*mtLSU*+*mtSSU* sequence dataset where 3–4 phylogenetic markers were obtained for every specimen provided phylograms with high support for most of the clades. As the Bayesian 50% majority-rule consensus tree had the same topology as that of the IQ-tree, both phylograms are combined in Figure 1.

Sequences of *Umbilicaria krascheninnikovii* were combined in a well-supported clade in all single markers (Figures S1–S4) and combined phylograms (Figure 1).

**Figure 2:**
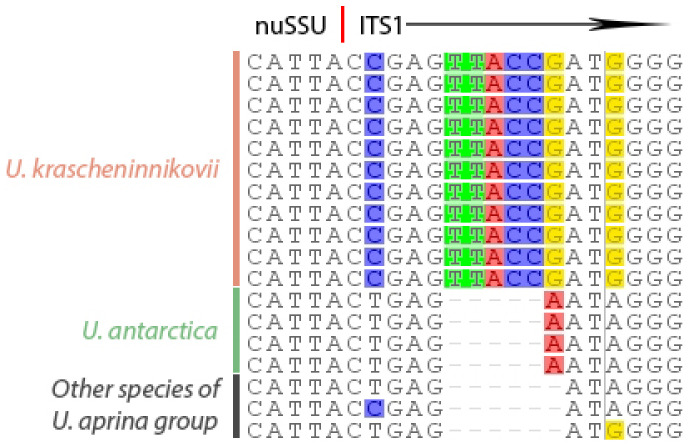
Alignment of nucleotide sequences within a final portion of small subunit of the nuclear ribosomal DNA (*SSU*) and started portion of internal transcribed spacer region (*ITS1*). Disagreements with the consensus sequence are highlighted.

The *ITS* sequences of *Umbilicaria krascheninnikovii* were slightly variable (1–4 residues pairwise). The hypervariable part of the *ITS1* of all sequences of *U. krascheninnikovii* contains the insertion TTACCG (Figure 2) between positions 5 and 6, which repeats the sequence of the three last positions of *nuSSU* and positions 1–3 of *ITS1* and was never observed within other species of Umbilicariaceae (the dataset in [31]). Sequences of *U. antarctica* had a single nucleotide A in the same position.

All sequences of *Umbilicaria antarctica* and *U. rhizinata* were combined in a well-supported clade. In contrast, *U. africana* and *U. formosana* appeared to be non-monophyletic. Sequences of *U. africana* from Africa and *U. formosana* from Yunnan clustered together with strong support in combined analyses (100% PP; 1.00 BS, Figure 1), while sequences of *U. africana* from the Southern Hemisphere, i.e., from the Antarctica and south of Chile, clustered between the most basal clade, *U. antarctica*, and the remaining sequences of the *U. aprina* group. Sequences of *U. formosana* from South Siberia clustered as a sister to *U. rhizinata*.

*Umbilicaria krascheninnikovii* is a sister to the clade containing *U. africana* from Africa and *U. formosana* from Yunnan. This relationship has rather strong support in *mtSSU*, *mtLSU*, and combined analyses (Figure 1, Figures S3 and S4). However, *U. krascheninnikovii* grouped with the South Hemispheric *U. aprina* in the *ITS* analyses (Figure S1) but without statistical support (63% PP; 0.60 BS).

*Umbilicaria polaris* clustered apart of *U. krascheninnikovii* and, generally, the *U. aprina* group. 

### 2.2. The Description of Umbilicaria krascheninnikovii

Because *Umbilicaria krascheninnikovii* has been misunderstood in the scientific literature for a long time, we present herein the full description of the species based on the type materials and our collection, including most of the existing herbarium specimens.

**Figure 3:**
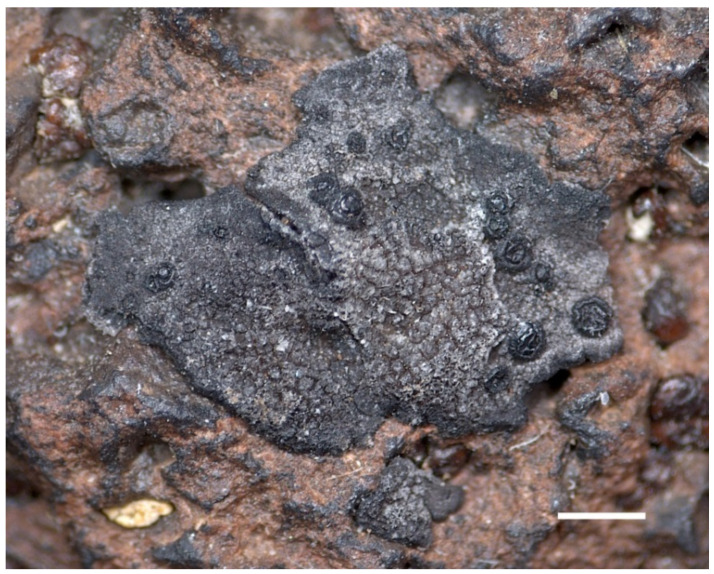
The fragment of the holotype of *Umbilicaria krascheninnikovii* (LE-L279). Scale = 1 mm.

**Figure 4:**
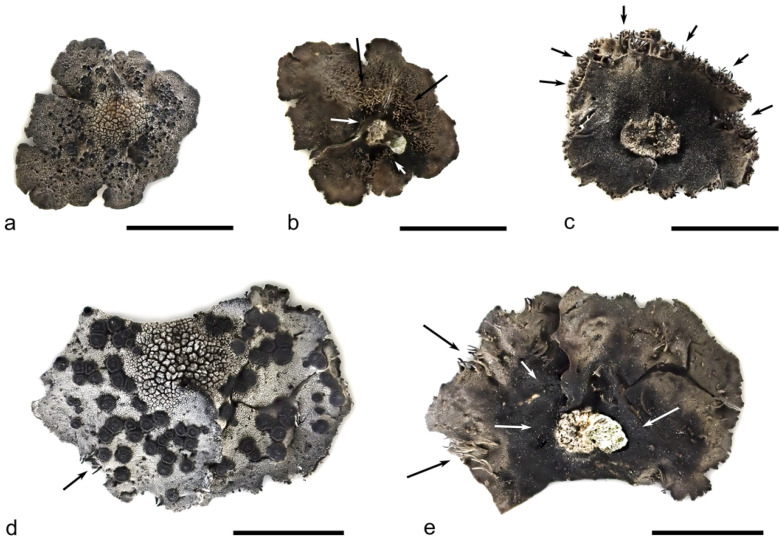
*Umbilicaria krascheninnikovii:* (**a**) Thallus upper surface with characteristic areolate center and numerous young apothecia; (**b**) thallus lower surface with rhizinomorphs and darker central part; (**c**) thallus lower surface with marginal rhizinomorphs (a rare trait); (**d**) mature specimen upper surface; (**e**) mature specimen lower surface. Black arrows—rhizinomorphs; white arrows—patches of thalloconidia. Scales: (**a**–**e**) = 5 mm.

**Figure 5:**
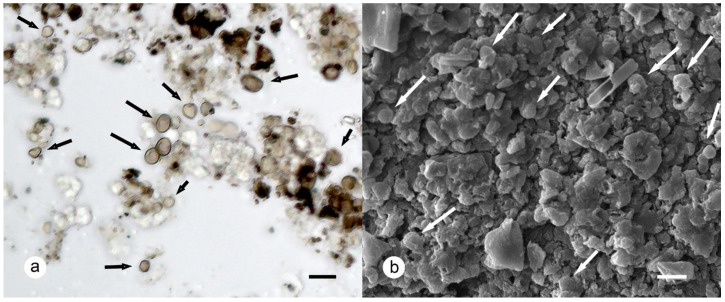
Thalloconidia of *Umbilicaria krascheninnikovii*: (**a**) light-brown thalloconidia (arrows) in water (holotype); (**b**) SEM of lower surface with scarce thalloconidia (arrows). Scales: (**a**,**b**) = 10 μm.

**Figure 6:**
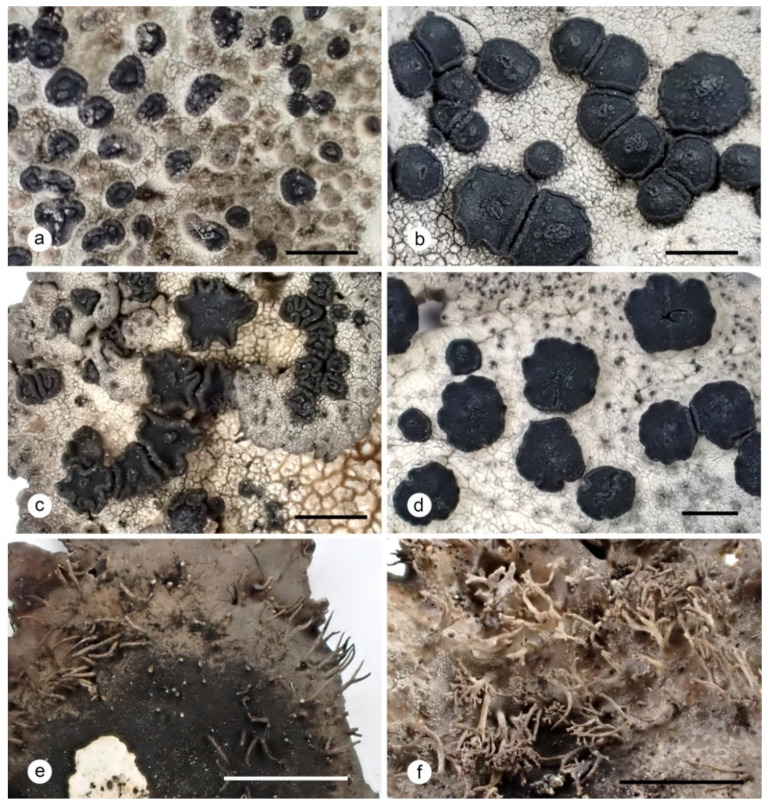
Variability of apothecia and rhizinomorphes of *Umbilicaria krascheninnikovii*: (**a**) apothecia protrunding through the upper cortex; laterally corticated apothecia; (**b**) leiodisc (upper left corner) and omphalodisc apothecia with one to several gyri; (**c**) lobate omphalodisc apothecia; (**d**) omphalodisc apothecia with fissures; (**e**) typical simple to one-branched rhizinomorphs; (**f**) branched rhizinomorphs. Scales: (**a**–**d**) = 1 mm; (**e**,**f**) = 5 mm.

**Figure 7:**
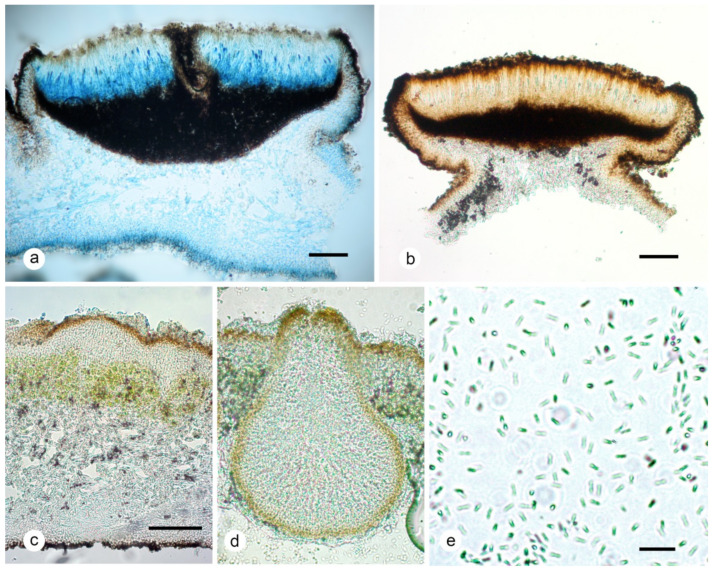
*Umbilicaria krascheninnikovii.* (**a**). Section of apothecium with immature asci in lactophenol cotton blue; (**b**) section of leiodisc apothecium showing black-brown hypothecium; (**c**) section of thallus; (**d**) pycnidia; (**e**) bacilliform conidia. Scales: (**a**–**c**) = 100 μm; (**d**) = 50 μm; (**e**) = 10 μm.

*Umbilicaria krascheninnikovii* (Savicz) Zahlbr. Cat. Lich. Univ. 10: 405, 1939. ≡*Gyrophora krascheninnikovii* Savicz, Izv. Imp. Bot. Sada Petra Velikogo 14 (1–2): 117, 1914. —Holotype: (Russia, Kamczatka), supra massam sulphuream ad rupes vulcanicas montis (“sopkae”) Krascheninnikovii (in ripis lacus Kronotzkoje) abundanter lecta, 1909, V. P. Savicz, No. 6412 (LE-L279!) ≡*Omphalodiscus krascheninnikovii* (Savicz) Schol. Nyt Mag. Naturvid. 75: 24, 1934 (Figure 3, Figure 4, Figure 5, Figure 6 and Figure 7).

=*Gyrophora hultenii* DuRietz, Arkiv for Bot. 22 (13): 14, 1929.—Type: (Russia), Syd-Kamchatka, Avachinskaya Sopka Volcano, c. 700 m, 31 July 1920, Hulten E. (Isotype, LE-L568!) ≡*Umbilicaria hultenii* (Du Rietz) Zahlbr. Cat. Lich. Univ. 8: 492, 1932.

Thallus of small size, 0.5–1.5(4.0) cm diam. and 0.2–0.5 mm thick, umbilicate, monophyllous, rigid; the margins first entire, orbicular, very rarely with marginal rhizinomorphs; later margins becoming incised with edges turned down or necrotic; upper surface minutely rimose to areolate; pale to dark grey, sometimes ochraceous, at margins sometimes with a brown tinge, at the central part over the umbilicus ascending, roughly areolate, with a thick cracked epinecral layer resembling angular crystals (Figure 4a,d); lower surface around the umbo dark brown to black with patches of thalloconidia (Figure 4b,e and Figure 5), remaining surface light brown to grey-brown or grey, darker towards the margins to dark grey, smooth to slightly rimose with scarce rhizinomorphs (Figure 4b,e); rhizinomorphs cylindrical or slightly flattened, unbranched to 1–2(–3) times dichotomously branched (Figure 6e,f); thalloconidia sometimes present on the lower surface at central part, making it appear darker, brown to black; simple, exceptionally one-septate, light brown to brown, spherical to ellipsoid (5.1–)5.5–6.2–6.8(–7.1) × (5.0–)5.1–5.5–6.0(–6.5) μm (*n* = 25) (Figure 5). Upper cortex paraplectenchymatous, brownish at the outer part and hyaline in the inner part, 32–88 μm thick; epicortex hyaline, paraplectenchymatous with an amorphous upper part, cracked, 15–65 μm thick; algal layer continuous, 45–89 μm thick, algae trebouxioid; medulla colorless, more or less loose, 65–210 μm thick; lower cortex scleroplectenchymateous, 32–68 μm thick, including a colorless inner layer and a brown outer layer, in the central part transforming into thalloconidia (Figure 7c). Apothecia common and abundant on upper surface except for the central part of thalli, 0.5–1.0(–2.0) mm in diam., at first sessile, sometimes protruding through and surrounded by the upper cortex, with a central gyrus, the latter with several sterile buttons (polyomphalodisc); some of apothecia lack sterile tissue at the center, with age, apothecia increase in size and become lobed (Figure 6a–d); epihymenium brown, 12.5–15.0 μm thick; hymenium hyaline 63–73 μm thick, hypothecium black brown, 35–45 μm thick; excipulum in the inner part yellowish and in the outer part brown; paraphyses septate, branched, 1.8 μm thick, sometimes slightly thickened at the tips, up to 2.0–2.5 μm; *asci* 45–65 × 12–17 μm, containing eight ascospores; ascospores hyaline, non-septate, (7.0–)8.0–9.1–10.1(–11.0) × (3.0–)3.7–4.5–5.3(–5.5) μm (*n* = 31), often immature (Figure 7a,b).

Pycnidia common at the periphery of the thalli, 150–220 μm in diam., with black prominent ostiole; wall light brown, 10 μm thick; pycnoconidia bacilliform, 3.2–4.0 × 1 μm (Figure 7d,e).

Chemistry. Thallus K–, C+ red, KC+ red, Pd–, UV–; gyrophoric (major) and lecanoric (minor) acids detectable by TLC.

**Diagnostic traits and variability**. Most species in the Umbilicariaceae consist of individuals that exhibit a series of developmental stages [64]. Similar to vascular plants, these ontogenetic stages can be roughly divided into juvenile, mature, and senescent stages. The juvenile stage is represented by thalli up to ca. 5 mm in diameter with entire margins, a light-gray upper surface with numerous warts with pycnidia and apothecia, and a light-brown lower surface with simple, same-colored rhizinomorphs without thalloconidia. Such specimens resemble *Umbilicaria rhizinata*, but juvenile specimens of the latter species rarely possess pycnidia and soon produce thalloconidia. Apothecia appear very soon, i.e., on very small thalli (Figure 3 and Figure 4a). At the same time or later, thalloconidia begin to develop on the lower side around the umbo. Mature specimens are described above (Figure 4). *Umbilicaria krascheninnikovii* resembles *U. rhizinata*, from which it differs in non-septate (vs. 3–4 cellular), sporadically produced (vs. always produced) thalloconidia and abundant apothecia. *Umbilicaria krascheninnikovii* is also close to *U. aprina*, from which *U. krascheninnikovii* differs in smaller thalli, light-brown thalloconidia that sometimes develop as patches on the lower surface, and scarce rhizinomorphs. The upper surface of senescent specimens becomes irregularly wrinkled and overgrown with crowded apothecia or with numerous initial pycnidia and apothecia. Individual apothecia increase in size up to 2 mm and become lobed (Figure S5). Apothecia of *Umbilicaria krascheninnikovii* are remarkably variable (Figure 6a–d). Leiodisc apothecia and apothecia with fissures are common for *U. virginis* Schaer., and lobate apothecia for *U. antarctica* [65]. But those species are only distantly related to *U. krascheninnikovii. Umbilicaria krascheninnikovii* stands out among other species of the *U. aprina* group, with fertility beginning at the early stages of its ontogeny. It differs from *U. polaris* in the small size of thalli, the presence of thalloconidia, and the absence of the reticulate pattern on the upper surface (see also Table 1).

**Figure 8:**
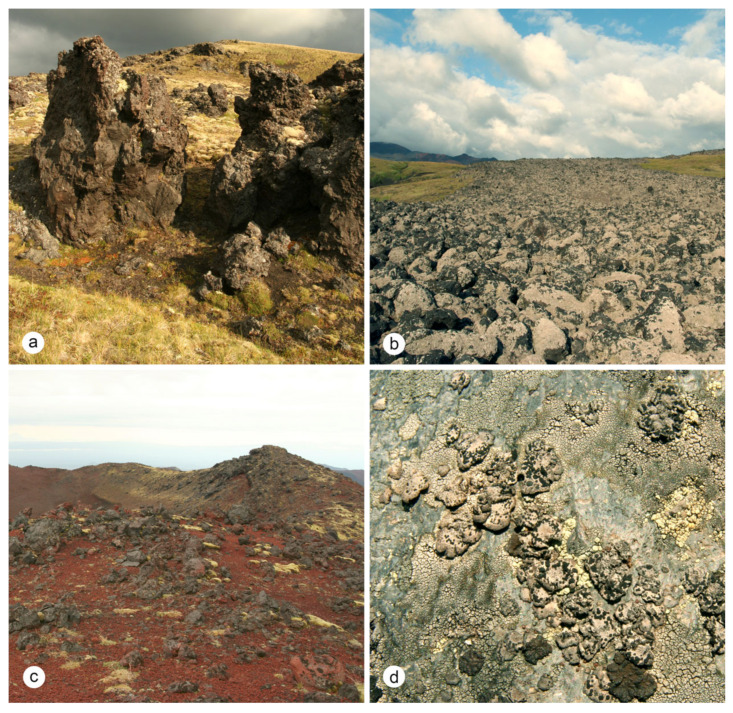
Typical habitats of *Umbilicaria krascheninnikovii* on SW slope of Tolbachik Volcano: (**a**) lava remnants in mountain tundra; (**b**) lava flow “1941” with pioneer vegetation dominated by *Stereocaulon vesuvianum* Pers.; (**c**) top of the side cone with pioneer vegetation. *U. krascheninnikovii* occurs on lava stones; (**d**) saxicolous lichen community with *U. krascheninnikovii* on volcanic bomb.

**Distribution and Ecology**. *Umbilicaria krascheninnikovii* grows on lava flows and boulders in a wide range of communities in open places at elevations from 370 to 1610 m a.s.l. in Kamchatka and Kurile and up to 3700 m s.s.l. in Japan (Figure 8 and Figure S6). 

*Umbilicaria krascheninnikovii* is locally common and widespread in central and south Kamchatka in areas of modern volcanism but unknown in north Kamchatka (Koryakia) in areas of ancient volcanism. We are not able to estimate the abundance of this species in Kurile Island and Japan since only herbarium collections from those islands have been studied (Figure S6, Table S2).

**Figure 9:**
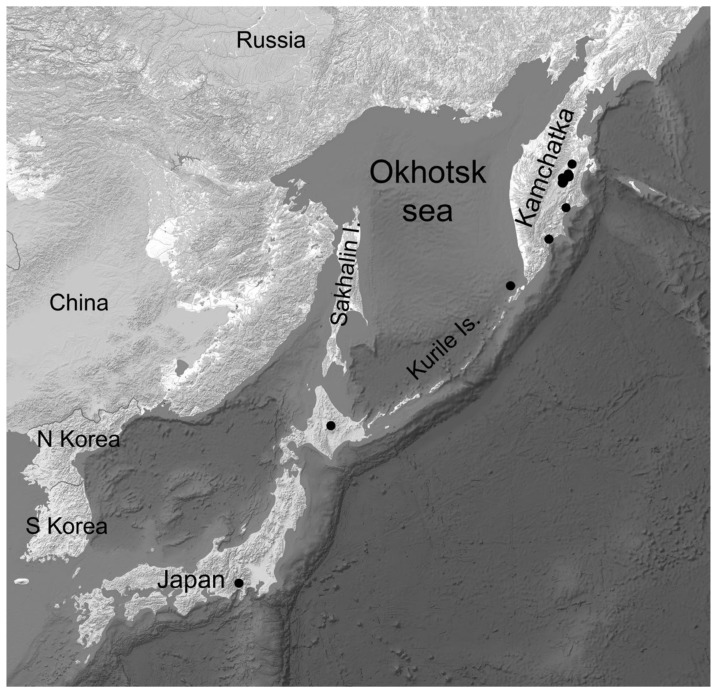
The known distribution of *Umbilicaria krascheninnikovii*.

According to our knowledge, *Umbilicaria krascheninnikovii* is endemic to East Asia, or more precisely, to Kamchatka, Kurile, and Japan (Figure 9). Wei and Jiang [36] presumed that the records of *U*. *krascheninnikovii* auct. non (Savicz) Zahlbr. may belong to *U*. *formosana*, but this applies mostly to Chinese material. Specimens of *Umbilicaria krascheninnikovii* from China in the herbarium HMAS were re-identified by the first author as *U*. *formosana*. According to the data of the first authors from many herbaria, most records of *U*. *krascheninnikovii* auct. non (Savicz) Zahlbr. outside East Asia belong to *U. polaris*.

Specimens of *Umbilicaria krascheninnikovii* are examined in Table S2.

### 2.3. Reconstruction of the Character Evolution in the Umbilicaria aprina Group

**Figure 10:**
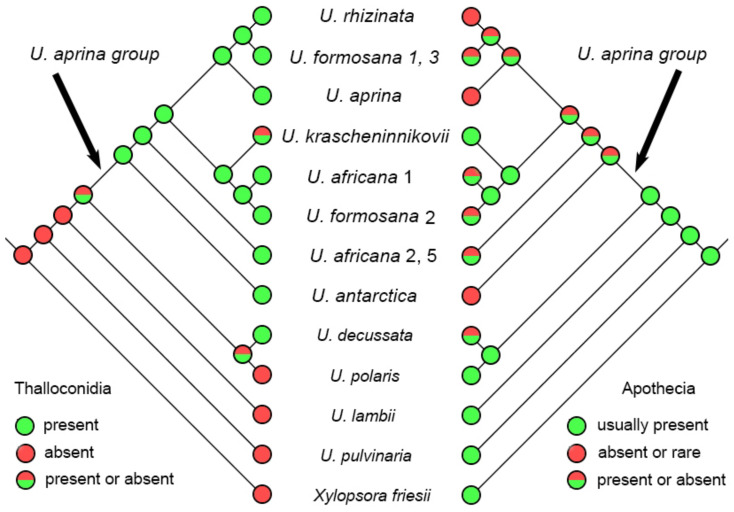
Reconstruction of the evolution of traits based on tree in Figure 1. **Left**: thalloconidia presence. **Right**: apothecia presence.

All species of the *Umbilicaria aprina* group possess thalloconidia on the lower surface (Figure 10). This trait is characteristic for the *U. aprina* group but not unique among *Umbilicaria*. Species of the *Umbilicaria aprina* may also produce apothecia. Sexual reproduction in *U. rhizinata* is unknown, and in *U. aprina* and *U. antarctica*, it is extremely rare. *U. africana* and *U. formosana* normally produce both thalloconidia and apothecia. *Umbilicaria krascheninnikovii* stands out among other species of the *U. aprina* group, commonly having apothecia but often lacking thalloconidia (Figure 10).

### 2.4. Reconstruction of the Biogeographic History in the Umbilicaria aprina Group

The models without the “+J” counterpart fit the data better than the models with the “+J” counterpart. The model BAYAREALIKE was selected as the best-fit model to explain the speciation and dispersal history of the *U. aprina* group, and this method allows the inclusion of potential dispersal events in the analysis (Table 2; AICc model weight = 0.70). Under the best-fit model BAYAREALIKE, at least 16 dispersal (range expansion) events and no extinction or vicariance event were inferred to form the current geographic pattern of species distribution in the *U. aprina* group. This was consistent with the results of the second-best-fit model (Table 2; AICc weight = 0.3); BAYAREALIKE +J showed exactly the same pattern with slightly lower probabilities at nodes. No nodes were inferred as “speciation within areas”, which is defined as two descendants with the same range as the ancestor, that is, in situ diversification. This fact suggests that long-distance dispersal does play a crucial role in the biogeography and speciation of the *Umbilicaria aprina* group. Alternatively, it is possible that molecular phylogenies are missing many speciation events—those that led to species that are extinct.

**Figure 11:**
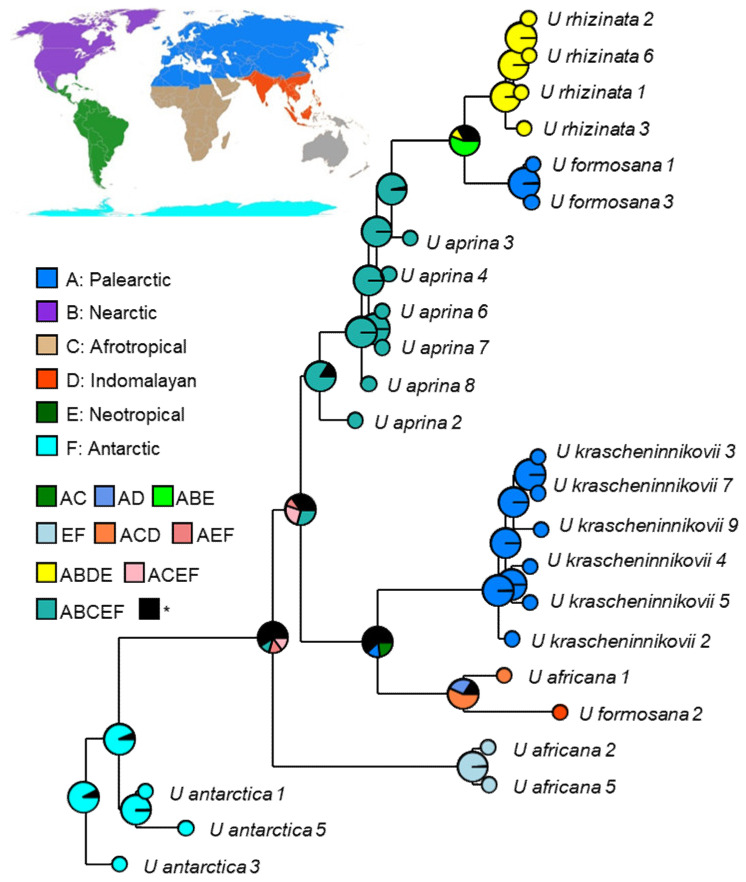
Current large-scale distribution and reconstructed ancestral distribution of species of the *Umbilicaria aprina* group based on the best-fit model (BAYAREALIKE) using BioGeoBEARS. Node colors reflect biogeographic designations (for species at tips) and ancestral area reconstructions (for internal nodes); areas less than 10% were hidden. * - not defined.

The analyses suggest that two species in East Asian areas within the Palearctic, i.e., *U. formosana* 1&3 and *U. krascheninnikovii*, speciated independently but both from widely distributed ancestors. Based on the BAYAREALIKE, the ancestors of *U. krascheninnikovii* likely inhabited the Palearctic and Afrotropical realms or wider—fewer probably from just the Palearctic (Figure 11). The current geographic distribution of *U. krascheninnikovii* in areas of modern volcanism in the Kamchatka Peninsula suggests that orogeny might have promoted the diversification the *Umbilicaria aprina* group and speciation of *U. krascheninnikovii*.

## 3. Discussion

### 3.1. Phylogeny of the Umbilicaria aprina Group

*Umbilicaria krascheninnikovii* in its more refined circumscription appears as a monophyletic species in all analyses. Despite the variability of such morphological traits such as thalloconidia presence, rhizinomorph presence and branching, sterile tissue development on apothecia, etc., we do not have any phylogenetic signal of infraspecific differentiation because these traits varied in one collection or even on one specimen. The phylogenetic position of *U. krascheninnikovii* within the *U. aprina* group was suggested in Davydov et al. [31] based on a few *ITS* and *mtLSU* sequences. In the present 3–4 marker analyses, the *U. krascheninnikovii* clade always has the terminal position. The close relationship of *U. krascheninnikovii* to *U. africana* and *U. formosana* is not obvious from morphology, with the latter two species having larger size, a reticulate pattern on the thallus upper surface, and 4–24 cellular thalloconidia in *U. africana* versus non-septate ones in *U. krascheninnikovii*. It is remarkable, however, that all three species often produce both ascospores and thalloconidia.

Species of the *Umbilicaria aprina* group reproducing mostly (*U. antarctica*) or exclusively (*U. rhizinata*) asexually are monophyletic, and almost no variations have been found in the ITS rDNA sequences of these species. This is consistent with their asexual reproduction, mainly producing genetic clones. *Umbilicaria antarctica* has a more restricted distribution, and its *ITS* and *mtSSU* sequences were studied in a relatively wide range of its distribution in Antarctica [66,67] with the same result. However, populations of cosmopolitan *U. rhizinata* from the Himalaya Mountains and South America have not been involved in molecular phylogenetic analyses yet, and our result therefore is valid only for the Holarctic populations of this species. Sequences of *U. aprina*, a species that reproduces mainly asexually but sometimes has apothecia, do not form a well-supported clade. Both *Umbilicaria africana* and *U. formosana*, species that reproduce both sexually and asexually, are apparently non-monophyletic and are grouped regionally: *U. africana* from the Northern and Southern Hemispheres and *U. formosana* from South Asia or East Asia and Siberia. We assume that such a pattern is a consequence of two modes of reproduction common in the *Umbilicaria aprina* group. Since the rates of recombination and speciation are usually higher in sexually reproducing species, the large distance and, consequently, the possible isolation among regions where *Umbilicaria africana* and *U. formosana* were sampled could lead to the divergence into (crypto-) species or genetically differentiated subpopulations of the same species. The species status of the identified lines, namely *U. africana* and *U. formosana,* should be critically evaluated using a wide range of samples.

Grebelnyi [68], summarizing a large number of observations about cloning in nature, concluded that genetic polymorphism obviously increases during sexual reproduction, but only a small part of the individuals of the species are really well adapted to the environment. Opposite, the clones resulting from asexual reproduction are genotypically homogenous and have negligible evolutionary potential in comparison with ancestral, bisexual populations. But a successful clone consists of individuals most adapted to the current conditions, which can spread over most of the area, and bisexual populations, despite their disproportionately large evolutionary possibilities, are often limited in distribution. Clonal reproduction is common in fungi and fungal-like organisms during invasion events [69], but truly asexual fungi are rare; most Ascomycota use both clonal and sexual reproduction during different stages of their lifecycle, depending on environmental conditions, substrate, and nutrient availability [70]. Despite energetic costs, sexual reproduction is still a common feature of lichens in extreme habitats and should generate novel genetic diversity within populations besides spore production [71]. Furthermore, sexual reproduction involving heterothallism provides a mechanism for promoting such genetic diversity within lichen populations [72]. The sexual breeding system (homothallic or heterothallic) was not studied in Umbilicariaceae.

Many groups of lichens include closely related taxa with contrasting reproductive modes: One taxon reproduces sexually and the other vegetatively. The evolutionary processes underlying such “species pairs” are still unknown [73]. In symbiotic organisms such as lichens, a second variable, i.e., the photobiont, has to be considered. The cloning of lichens can be carried out by asexual reproduction by non-lichenized (e.g., thalloconidia) or lichenized (e.g., soredia and isidia) dispersal units. In *Umbilicaria*, clonal reproduction by thalloconidia still requires the finding of a compatible photobiont, as in dispersal by ascospores.

Apparently, the evolution of species within the *Umbilicaria aprina* group includes sexual stages generating genetic diversity, dispersal, and invasion of successful clones into the new territory. The subsequent isolation of sexually reproducing lines leads to the accumulation of genetic differences or, under certain conditions, to the formation of new species. *Umbilicaria krascheninnikovii* may represent such an example of a species adapted to specific conditions in volcanic regions. Species that reproduce mainly asexually, in our case by thalloconidia, in the presence of dispersal vectors colonize similar habitats sometimes at considerable distances, which is true, for example, for *U. antarctica, U. africana, U. aprina,* and *U. rhizinata*. Umbilicaria antarctica is the most basal species in the phylogeny of *U. aprina*. It is unlikely that *U. antarctica* is an ancestral taxon in the group *U. aprina* because it is too specialized for certain conditions and almost lacks sexual reproduction. Probably, its asexual reproduction, successful in the harsh conditions of Antarctica, really preserves ancestral genotype lines within the *U. aprina* group. *Umbilicaria krascheninnikovii* forms a terminal branch and is therefore considered by us as a neo-endemic species.

### 3.2. Phenotypic Traits and Endemism

A priori, we assumed *Umbilicaria krascheninnikovii* to be paleo-endemic because it combines ancestral traits such as a predominance of sexual reproduction and immaturity of thalloconidia, which we interpreted as the initial stages of evolution of thallogenesis in *U. aprina* group. Moreover, volcanism has been active since the early stages of Earth’s history and could be a factor in the speciation for a common ancestor of the *U. aprina* group. However, this hypothesis was not supported by the phylogenetic and biogeographic analyses (Figure 1, Figure 10 and Figure 11).

Mature thalli of *Umbilicaria krascheninnikovii* are usually fertile, often with crowded apothecia covering the upper surface, except for the central part and the peripheral rim. Fertility is a relatively rare trait for species of *Umbilicaria* that produce thalloconidia [31,58]. Although apothecia are common, the mature ascospores in *U. krascheninnikovii* can be rarely observed. On the other hand, thalloconidia of *U. krascheninnikovii* also often look immature. Typically, thalloconidia during maturation become thick-walled and pigmented, i.e., brown to dark brown or black-brown [74], and look like black farinose patches on the lower surface of thalli. Thalloconidia of *U. krascheninnikovii* are often colorless to light brown (Figure 5a), and the lower surface rarely has farinose patches because thalloconidia are scarce (Figure 5b).

Apparently, sexuality increased and thalloconidiogenesis decreased in *Umbilicaria krascheninnikovii* relative to the remaining representatives of the *U. aprina* group (Figure 10). It could be explained by functional coupling of traits: For species having thalloconidia or lichenized propagules, the significance of propagation by ascospores is decreased, and vice versa [31]. Because thalloconidia production is a plesiomorphic trait in the *U. aprina* group, and fertility is more or less common only for *U. africana* and *U formosana*, it can be assumed that *U. krascheninnikovii* is switching from asexual reproduction by thalloconidia to sexual reproduction by ascospores. The biological sense of returning to sexual reproduction may be in increasing recombination because “the evolutionary potentialities of an organism depend upon how great a variety of gene combinations it is capable of producing” [75] and can be considered as adaptation to specific conditions in active volcanic regions where rock surfaces are periodically catastrophically transformed by volcanic activity, and most communities do not reach the climax for a long time and remain in pioneer stages of succession. Related taxa, namely *Umbilicaria aprina* and *U. rhizinata*, both reproduced exclusively by thalloconidia, are also collected in Kamchatka but, unlike *U. krascheninnikovii*, occur extremely rarely. 

Thus, the combination of sexual and asexual reproduction provides adaptive advantages in changing environmental conditions. The endemic distribution of *U. krascheninnikovii* can be a consequence of the possible lower vitality of an observed significant proportion of immature thalloconidia and immature ascospores, making it less likely to spread over long distances.

### 3.3. East Asian Distribution Pattern and Kamchatian Endemism

The *Umbilicaria aprina* group unites species characteristic to harsh environments in polar and high mountain regions worldwide but with different patterns of distribution. *Umbilicaria africana*, *U. aprina*, and *U. rhizinata* are distributed on several continents both in the Northern and Southern Hemispheres; *U. antarctica* and the phylogenetically related taxon or ecomorph *U. kappenii* (see [67]) are endemic to the Antarctic region [54], *U. formosana* is an East Asian species [76], and *U. krascheninnikovii* is a species with the most restricted distribution in East Asia (Figure 9).

The Eastern Asiatic region is known as one of the diversity and endemism hotspots in the world and the richest floristic region within the Holarctic [77,78,79] as well as one of the centers of species diversity and endemism of *Umbilicaria* [36]. A lot of East Asian species also occur in the South Siberian mountains at the northern edges of their ranges [80] and Tibet in the south [81]. This is applicable for the distribution of species of *Umbilicaria* such as *U. formosana*, *U. kisovana* (Asahina) Zahlbr., *U. orientalis* Davydov, and *U. squamosa* J. C. Wei & Y. M. Jiang [38,82]. There are only a few endemics of the Eastern Asiatic region with restricted distribution, and they have different origins. *Umbilicaria esculenta* (Miyoshi) Minks and *U. loboperipherica* J. C. Wei et al. represent species of humid mountain forests [83,84]. *Umbilicaria pulvinaria* (Savicz) Frey is restricted in its distribution to seaside mountains in Kamchatka and Sakhalin [85,86].

Views on the endemism of Kamchatian biota have changed in course of its investigation (e.g., [43,44,45,46,87,88,89,90]). Previously, it was generally believed that since Kamchatka Peninsula is biogeographically isolated from the mainland, its biota is highly specific and has many endemic species, most of which are concentrated around volcanoes and hot springs [91]. However, subsequent studies have shown that this pattern is, at least, not universal. Some vertebrates, e.g., freshwater fish, are highly endemic in Kamchatka [92,93]. At the same time, the diversity of organisms inhabiting terrestrial ecosystems appeared to be relatively impoverished and not highly specific, which can be explained by the geological history of the region, including Pleistocene glaciation [91,94,95]. For vascular plants, almost no connection between endemism and volcanic activity has been revealed [91]. The same is true for lichens; not a single species has been found to be endemic to Kamchatka. There are two other *Cladonia* species associated with thermal fields in Kamchatka, namely *Cladonia vulcani* Savicz and *C. granulans* Vainio, but both of them are distributed more widely [96]. The third “endemic”, *Cladonia favillicola* Trass [87], was synonymized with *C. botrytes* (K. G. Hagen) Willd. [97]. However, for larger regions that include Kamchatka as a part (such as Beringia, the north of the Russian far east, and the North Pacific), a high level of endemism has been shown for different groups of organisms, (e.g., [94,98,99,100]). In case of lichens, a few species in addition to *U. krascheninnikovii* and *U. pulvinaria* have restricted distribution. For example, *Ochrolechia subplicans* (Nyl.) Brodo subsp. *hultenii* (Erichs.) Brodo [44,101,102], *Polycauliona comandorica* Himelbrant et al. [103], *Cladonia nipponica* Asahina, *C. pseudoevansii* Asahina, *Stereocaulon apocalypticum* Nyl., *S. saviczii* DR. [20], and some others are supposed to be endemic to Beringia. Probably, there are also undescribed endemic species in understudied groups, such as crustose saxicolous genera. However, for other lichens previously considered endemic, e.g., “Amphiberingian” [104], new localities outside the North Pacific are currently known. 

Thus, we are not aware of other lichen species with the same range as *Umbilicaria krascheninnikovii*, but given the unusual ecological preferences of this epilithic species, its distribution area covering regions of active volcanism in East Asia seems plausible.

## 4. Materials and Methods

### 4.1. Sampling

We collected fresh material of *U. krascheninnikovii* and other species from the *U. aprina* group, i.e., *U. aprina*, *U. formosana*, and *U. rhizinata.* Freshly collected specimens were deposited in the lichen herbarium of V. L. Komarov Botanical Institute (LE) and the South-Siberian Botanical Garden of Altai State University (ALTB). The type and other lichen materials were examined from herbaria ALTB, H, HMAS, LE, LECB, M, MAG, O, TNS, UHU, VLA, and the personal collections of the authors.

We used sequences obtained from GenBank exclusively from specimens we had previously studied morphologically, anatomically, and chemically. Details of the materials and GenBank accession numbers are presented in Table S1.

The geographical range of sampled specimens was as follows: *Umbilicaria africana* (5 specimens): Ethiopia, south Chile, east Antarctica; *Umbilicaria antarctica* (5 specimens): west Antarctica and King George Island; *Umbilicaria aprina* (10 specimens): Arctic—Svalbard, Franz Josef Land, Sverdrup Island, Continental Asia—Buryatia; *Umbilicaria formosana* (3 specimens): China—Yunnan and the far east of Russia—Primorye Territory; *Umbilicaria krascheninnikovii* (11 specimens): Kamchatka Peninsula; *Umbilicaria rhizinata* (12 specimens): Continental Asia—Altai Mts., Buryatia, Kamchatka Peninsula, North America—Alaska, Subarctic—Putorana Plateau, and the Magadan Region (see also Table S1 and Figure 1 and Figure S1).

### 4.2. Morphology and Anatomy

Morphological observations were made using a dissecting microscope. Cross-sections were cut by hand with a razor blade and observed after mounting in water using a stereomicroscope (Zeiss Stemi 2000-C) and a compound microscope (Zeiss Axio Lab.A1). Measurements of thalloconidia are presented as follows: (smallest value recorded–) (x − SD) − x − (x + SD) (–largest value recorded), where x is the (arithmetic) sample mean, and SD is the sample standard deviation. The two extreme values are given to the nearest 0.5 µm and the sample mean to the nearest 0.1 µm. We used a scanning electron microscope (SEM) to visualize thalloconidia. Microphotographs were obtained using a Carl Zeiss EVO MA 10 SEM. The samples were dried in air and fixed on aluminum stubs with double-sided sticky film, and then, gold was sprayed on them.

### 4.3. Chemical Analyses

Secondary products were analyzed by applying standard thin-layer chromatography techniques [105] using solvents A, B, and C.

### 4.4. DNA Extraction, Amplification, and Sequencing

Three to five apothecia or central thallus sections of up to 15 mm^2^ were excised under a magnifying lens and transferred to sterile 1.5 mL reaction tubes. Rhizinomorphs were removed to avoid potential contamination with DNA from endolichenic fungi. The samples were frozen in liquid nitrogen and powderized in the tubes using sterile pestles. 

The DNeasy Plant Mini Kit (Qiagen, Hilden, Germany), ChargeSwitch gDNA Plant Kit (Invitrogen, Waltham, MA, USA), diaGene Plant Kit (Dia-M, Moscow, Russia), or DiamondDNA Plant Kit (ABT, Barnaul, Russia) were used for DNA extraction as recommended by the manufacturers.

To test the phylogenetic relationships within the *Umbilicaria aprina* group, the internal transcribed spacer region of nuclear ribosomal DNA (*ITS*), the large subunit of the mitochondrial ribosomal DNA (*mtLSU*), the small subunit of the mitochondrial ribosomal DNA (*mtSSU*), and RNA polymerase II between six and seven conserved parts (*RPB2*) were amplified in a single reaction from freshly collected specimens as well as from frozen DNA extracts. Primers and cycling conditions for amplification of all genes are listed in Table 2. The same primers were used for sequencing. The program Geneious 6.0 (Biomatters Ltd., Auckland, New Zealand) was used for assembling sequence reads and datasets. Consensus sequences were compiled from double-stranded sequenced parts of the sequences. Sequences were aligned with those of species of the *U. aprina* group from the most comprehensive phylogenetic study of *Umbilicaria* by Davydov et al. [31]. To improve the resolution of the phylogenetic analyses, we added the *mtSSU* DNA marker along with the *ITS*, *mtLSU*, and *RPB2* used previously [31]. This marker showed phylogenetic signals on the species level in other phylogenetic works with Umbilicariaceae [26,68].

### 4.5. Sequence Alignment and Phylogenetic Analyses

All newly obtained sequences as well as sequences obtained during our previous study of Umbilicariaceae phylogeny [31], representing species of the *U. aprina* group as well as *U. decussata*, *U. polaris*, *U. pulvinaria*, and *U. lambii* Imshaug, were combined into datasets; *Xylopsora friesii* (Ach.) Bendiksby & Timdal was used as the outgroup. Selection of an outgroup was based on the studies of Wedin et al. [106], Bendiksby and Timdal [30], and Davydov et al. [31], in which *Xylopsora* formed the sister clade to *Umbilicaria*. GenBank Accession numbers are provided in Table S1. Four single-gene datasets containing the sequences listed in Table S1 were compiled and aligned using the MAFFT algorithm [107]. Introns in the *mtSSU* and *mtLSU* sequences were manually removed from alignments. Before combining sequences into a joint *ITS*+*RPB2*+*mtLSU*+*mtSSU* data matrix, the unambiguously alignable regions were used to calculate single-marker phylograms (Figures S1–S4) using the online version of IQ-TREE [108,109], which were tested for conflicts among datasets. Because the cladograms were similar regarding well-supported (BS ≥ 70%) clades and therefore lacked conflicts, sequences were combined into one matrix consisting of 2736 sites and used for maximum likelihood and Bayesian analyses. 

A heuristic search for the maximum likelihood (ML) bootstrap tree with simultaneous inference of the optimal partitioning scheme and substitution models for each data partition was performed using the online version of IQ-TREE [108,109], suggesting nine initial partitions (*ITS1*; *5.8S* rDNA; *ITS2*; *mtSSU*; *mtLSU*; *RPB2* 1st, 2nd, and 3rd codon positions; and intron within *RPB2*). Branch lengths were assumed to be equal for all partitions. Branch support was estimated with the ultrafast bootstrap algorithm [110] based on 1000 bootstrap replicates and using a maximum of 1000 iterations and a minimum correlation coefficient of 0.99 as a stopping rule. 

To provide additional support for our phylogenetic reconstruction, we ran a Bayesian analysis. We used the Markov chain Monte Carlo (BMCMC) method [111] implemented in MrBayes 3.2.3 [112] to infer phylogenetic trees applying the partitioning scheme inferred with IQ-TREE and slightly simplified substitution models inferred by PartitionFinder, ver. 1.1.1 [113], because most of the models inferred by IQ-TREE are not implemented in MrBayes (Table 3).

We applied these substitution models: a variable rate prior and an unconstrained exponential branch length prior, with a mean of 2.57. The mean of the branch length prior was calculated based on ML tree reconstructions using the procedure described by Ekman and Blaalid [114]. Three parallel analyses each with six incrementally heated chains using the default heating factor of 0.2 were run for 50 million generations, and every 200th generation was sampled. The first 50% of the trees were discarded as burn-in and a 50% majority rule consensus tree calculated from the remaining trees of the three runs with the sumt command implemented in MrBayes 3.2.3. Newly generated DNA sequences were uploaded to GenBank.

**Table 3:** Summary of data partitions and substitution models used for phylogenetic inference.

Name	*ITS1*	*5.8S rRNA*	*ITS2*	*RPB2*	*RPB2* Intron	*mtLSU*	*mtSSU*
Position	1–175	176–333	334–486	487–1175	1176–1212	1213–1949	1950–2736
PCR Settings							
Primers	ITS 1F-5′/ITS 4-3′ ITS 1F-5′/LR3-3′			RPB2-980F-5′/fRPB2-7cR-3′		ML 3-A-5′/ ML 4-A-3′	mrSSU1-5′/MSU7-3′
References	[115,116,117]			[118,119]		[120]	[121,122]
Datasets							
Alignment length (full)	180	158	153	689	36	1374	803
Alignment length (without ambiguous regions)	175	158	153	689	36	736	787
Polymorphic sites	39	7	19	139	18	111	79
Nucleotide diversity π	0.03508	0.00327	0.04473	0.05463	0.12607	0.02256	0.01720
Number of sequences (ingroup):	47	47	47	13	13	41	35
Substitution model (IQ-TREE)	TNe+G4	K2P+I	TNe+G4	pos.1: TN+F pos.2: F81+F pos.3: HKY+F+I	TNe	HKY+F+I	HKY+F+I
Substitution model (MrBayes)	K80+G	K80+I	K80+G	pos.1: K80+I pos.2: K80+I pos.3: HKY+I	HKY+I	HKY+G	HKY+G

### 4.6. Reconstruction of the Character Evolution

Character evolution was reconstructed using Mesquite ver. 3.2 [123]. Modules “Trace Character Over Trees” and “Reconstruct Ancestral States” with the parameter “parsimony reconstruction of ancestral states” were used to identify plesiomorphies and apomorphies.

### 4.7. Reconstruction of the Biogeographic History

Biogeographic history of the *Umbilicaria aprina* group was inferred using the BioGeoBEARS package implemented in RASP v4.3 [124] in comparison to the test model and using the MrBayes-generated phylogenetic tree without outgroups as input. Since the majority of the species of the *U. aprina* group has large areas of distribution, we used large-scale biogeographical analyses using six biogeographic realms: (A) Palearctic, (B) Nearctic, (C) Afrotropical, (D) Indomalayan, (E) Neotropical, and (F) Antarctic. Maximum range size was set to five because *U. aprina* occur in five of six biogeographic areas. An additional jump parameter (+J) was also used because this parameter can reveal founder-event speciation, which may result from long-distance dispersal and subsequent colonizing lineage divergence [125,126]. The null hypothesis that with J and without J conferred equal likelihoods on the data. Therefore, six models, namely the dispersal–extinction–cladogenesis (DEC) [127], an ML version of the dispersal–vicariance analysis (DIVALIKE) [128], and a version of the Bayesian analysis of historical biogeography for discrete areas (BAYAREALIKE) [129] as well as the corresponding “+J” models, were fitted using the corrected Akaike information criterion and Akaike weights to obtain the most suitable model for ancestral range reconstruction.

## Figures and Tables

**Table 1:** The diagnostic characters of *Umbilicaria krascheninnikovii* and morphologically similar species.

	*U. krascheninnikovii*	*U. rhizinata*	*U. aprina*	*U. formosana*	*U. polaris*
Thallus size	Small	Small	Medium	Medium	Medium
Upper surface center	Elevated, with rough “crystals” of necral layer, newer reticulate	Elevated, pruinose to areolate, newer reticulate	Elevated or depressed or weakly ridded, pruinose	Elevated or depressed, with reticulate pattern, pruinose	With reticulate pattern and rough “crystals” of necral layer
Apothecia	Common and abundant	Unknown	Rare	Common	Common and abundant
Thalloconidia	Eventually present, light brown to brown, non-septate	Common, brown, (1–2)3–4 cellular	Common, brown, non-septate	Common, brown, non-septate	Absent
Rhizinomorphs	Scarce or absent	Absent or scarce	Numerous	Absent, often with marginal bristles	Absent or scarce

**Table 2:** Statistical comparison of six different models using BioGeoBEARS and the selection of the best-fit model (BAYAREALIKE) for ancestral area reconstruction. Six models were compared using the corrected Akaike information criterion (AICc) weight method. The best model is highlighted in bold.

	Log Likelihood	Number of Parameters	AICc	AICc Model Weight
DEC	−105.8	2	216.1	1.2 × 10^−30^
DEC+J	−88.54	3	184.2	1.1 × 10^−23^
DIVALIKE	−113.4	2	231.3	6.3 × 10^−34^
DIVALIKE+J	−91.56	3	190.3	5.1 × 10^−25^
**BAYAREALIKE**	**−37.28**	**2**	**79.12**	**0.70**
BAYAREALIKE+J	−36.85	3	80.85	0.30

## Data Availability

The DNA sequence alignments were deposited at TreeBase (http://purl.org/phylo/treebase/phylows/study/TB2:S31004 (accessed on 5 December 2023)). Newly obtained sequences are deposited in the GenBank; for accession numbers, see Table S1.

## Supplementary Materials

The following supporting information can be downloaded at: https://www.mdpi.com/article/10.3390/plants13050729/s1, Figure S1: Phylogeny of *Umbilicaria aprina* group based on maximum likelihood analysis with IQ-TREE algorithm using the internal transcribed spacer region (ITS) sequences. The species names and GenBank accession numbers are indicated. The number in each node represents bootstrap support (BS) and posterior probability (PP). Branch lengths represent the estimated number of substitutions per site assuming the respective models of substitution. An exception is the branch with a black dot, which was shortened to reduce the overall figure size. Figure S2: Phylogeny of *Umbilicaria aprina* group based on maximum likelihood analysis with IQ-TREE algorithm using the large subunit of the mitochondrial ribosomal DNA (*mtLSU*) sequences. The species names and GenBank accession numbers are indicated. The number in each node represents bootstrap support (BS) and posterior probability (PP). Branch lengths represent the estimated number of substitutions per site assuming the respective models of substitution. An exception is the branch with a black dot, which was shortened to reduce the overall figure size. Figure S3: Phylogeny of *Umbilicaria aprina* group based on maximum likelihood analysis with IQ-TREE algorithm using the small subunit of the mitochondrial ribosomal DNA (*mtSSU*) sequences. The species names and GenBank accession numbers are indicated. The number in each node represents bootstrap support (BS) and posterior probability (PP). Branch lengths represent the estimated number of substitutions per site assuming the respective models of substitution. An exception is the branch with a black dot, which was shortened to reduce the overall figure size. Figure S4: Phylogeny of *Umbilicaria aprina* group based on maximum likelihood analysis with IQ-TREE algorithm using the RNA polymerase II (*RPB2*) gene partial sequences. The species names and GenBank accession numbers are indicated. The number in each node represents bootstrap support (BS) and posterior probability (PP). Branch lengths represent the estimated number of substitutions per site assuming the respective models of substitution. Figure S5: Typical habitats of *Umbilicaria krascheninnikovii* in Kamchatka Peninsula and Kurile Island. (A) SE slope of Kluchevskaya Sopka Volcano; (B) side (parasitic) cone and lava stream Apakhonchich on the SE slope of Kluchevskaya Sopka Volcano; (C) Ushkovsky Dale (foreground), volcanoes Kluchevskaya Sopka (left) and Kamen (right); (D) side cone on the SW slope of Ushkovsky Volcano; (E) N slope of Ostry Tolbachik Volcano; (F, G) side cones on the SW slope of Tolbachik Volcano. *Umbilicaria krascheninnikovii* in such localities can be found on lava and volcanic bombs; (H) Alaid Volcano in the Atlasova Island. Figure S6: Variability of senescent specimens of *Umbilicaria krascheninnikovii*. (A) Upper surface of small-sized senescent thallus with crowded apothecia; (B) entirely black lower surface; (C) upper surface of senescent thallus overgrown with apothecia; (D) irregularly wrinkled upper surface of small-sized senescent thallus with immature apothecia and pycnidia; (E) light-colored lower surface; (F) rather large senescent thallus without mature apothecia but with numerous black dots representing immature apothecia or/and pycnidia; (G) uncommonly large senescent thallus with large lobate apothecia; (H) lower surface with scarce rhizinomorphs. Table S1: Lichen samples used in this study, including voucher information and GenBank accession numbers. Table S2: Examined specimens of *Umbilicaria krascheninnikovii*.
