# Supplementary material for: Multilocus Molecular Phylogeny of the Umbilicaria aprina Group (Umbilicariaceae, Lichenized Ascomycota) Supports Species Level and Neo-Endemic Status of Umbilicaria krascheninnikovii
Source: Plants (Basel). 2024 Mar 4;13(5):729. doi: 10.3390/plants13050729 (PMC10933792; doi:10.3390/plants13050729)
Supplement: Supplementary file 1 [file plants-13-00729-s001.zip › plants-2783699-supplementary.pdf]

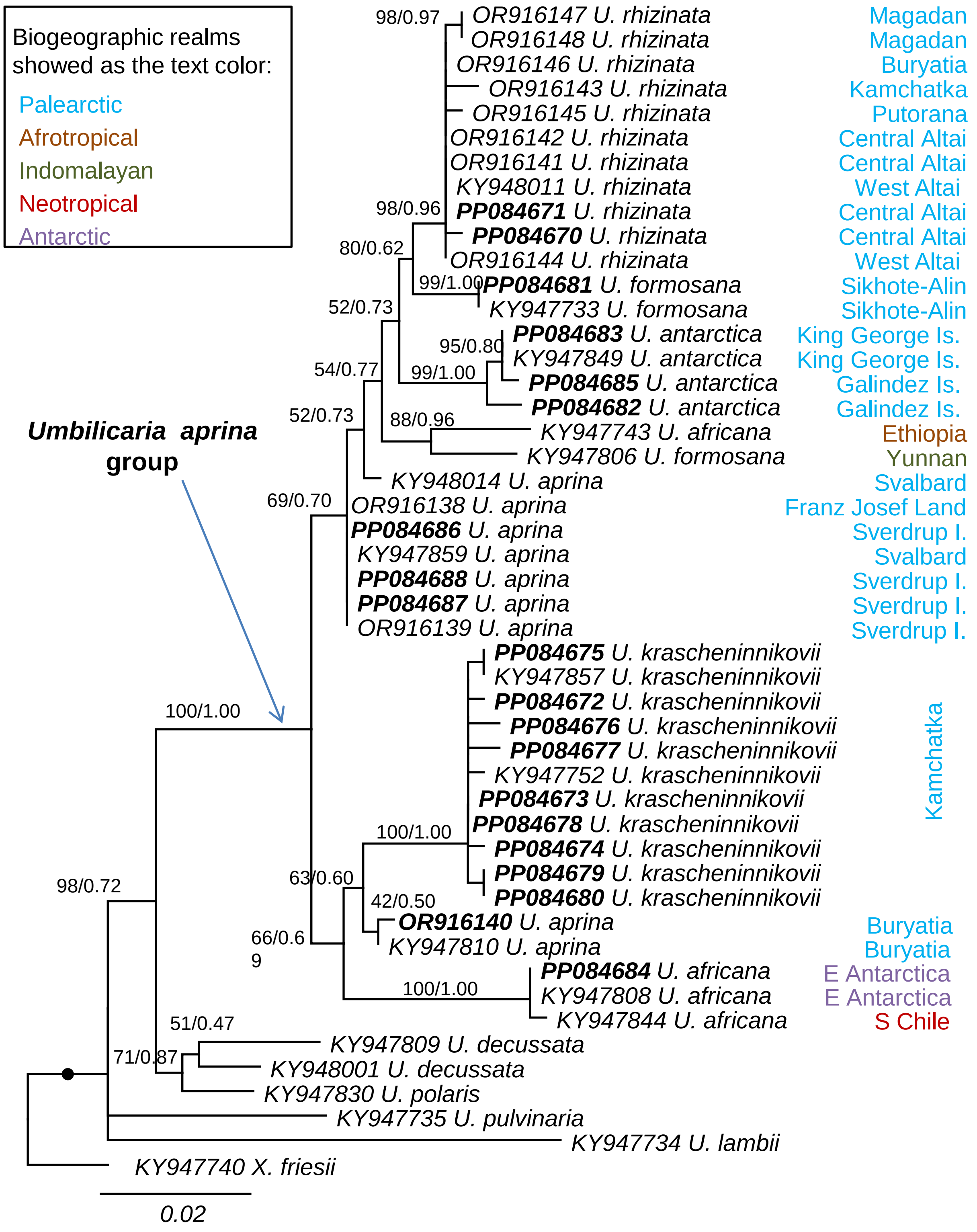

Figure S1. Phylogeny of *Umbilicaria aprina* group based on Maximum Likelihood analysis with IQ-TREE algorithm using the internal transcribed spacer region (ITS) sequences. The species names and GenBank accession numbers are indicated. The number in each node represents bootstrap support (BS) and posterior probability (PP). Branch lengths represent the estimated number of substitutions per site assuming the respective models of substitution. An exception is the branch with a black dot, which was shortened to reduce the overall figure size.

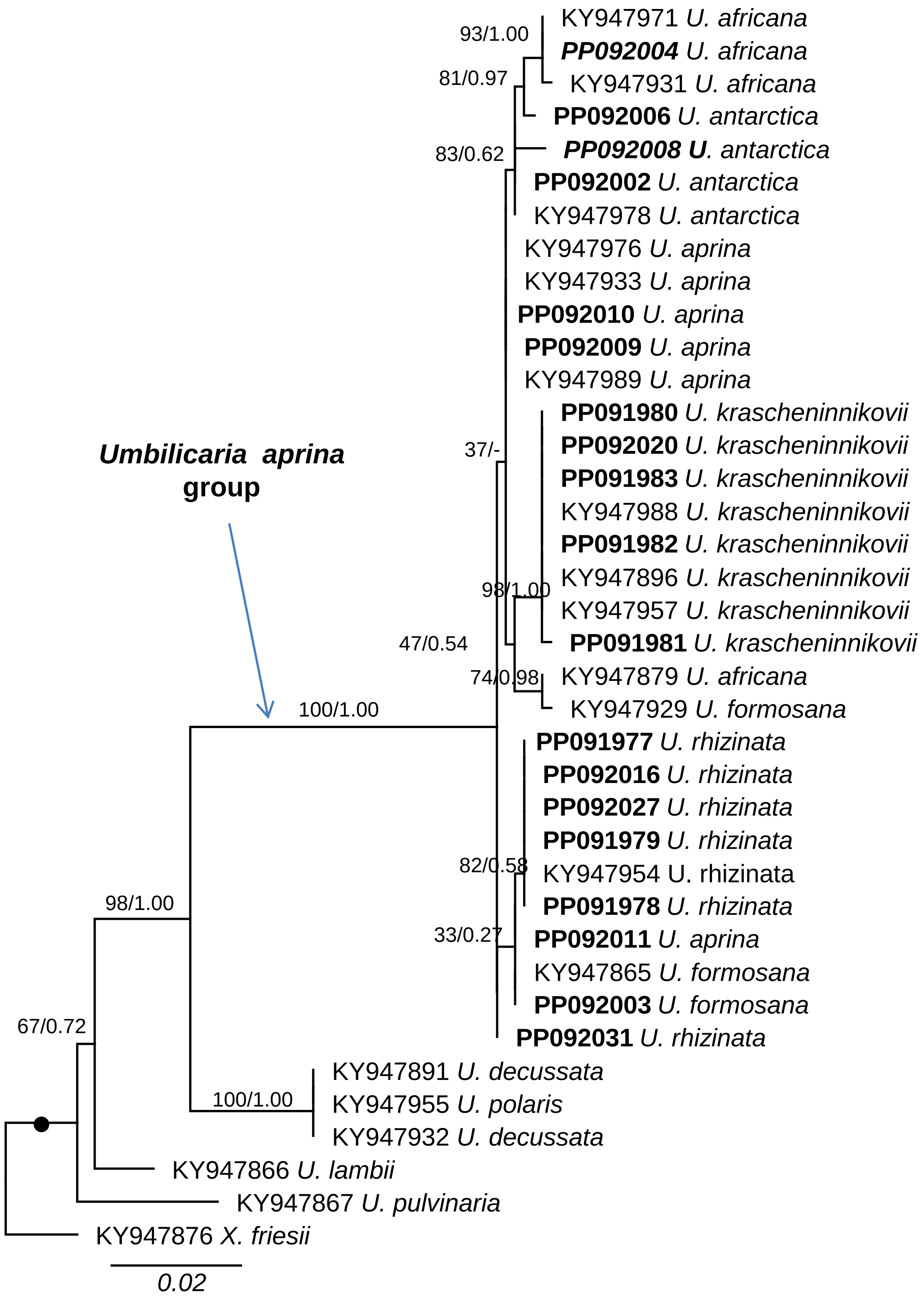

Figure S2. Phylogeny of *Umbilicaria aprina* group based on Maximum Likelihood analysis with IQ-TREE algorithm using the large subunit of the mitochondrial ribosomal DNA (mtLSU) sequences. The species names and GenBank accession numbers are indicated. The number in each node represents bootstrap support (BS) and posterior probability (PP). Branch lengths represent the estimated number of substitutions per site assuming the respective models of substitution. An exception is the branch with a black dot, which was shortened to reduce the overall figure size.

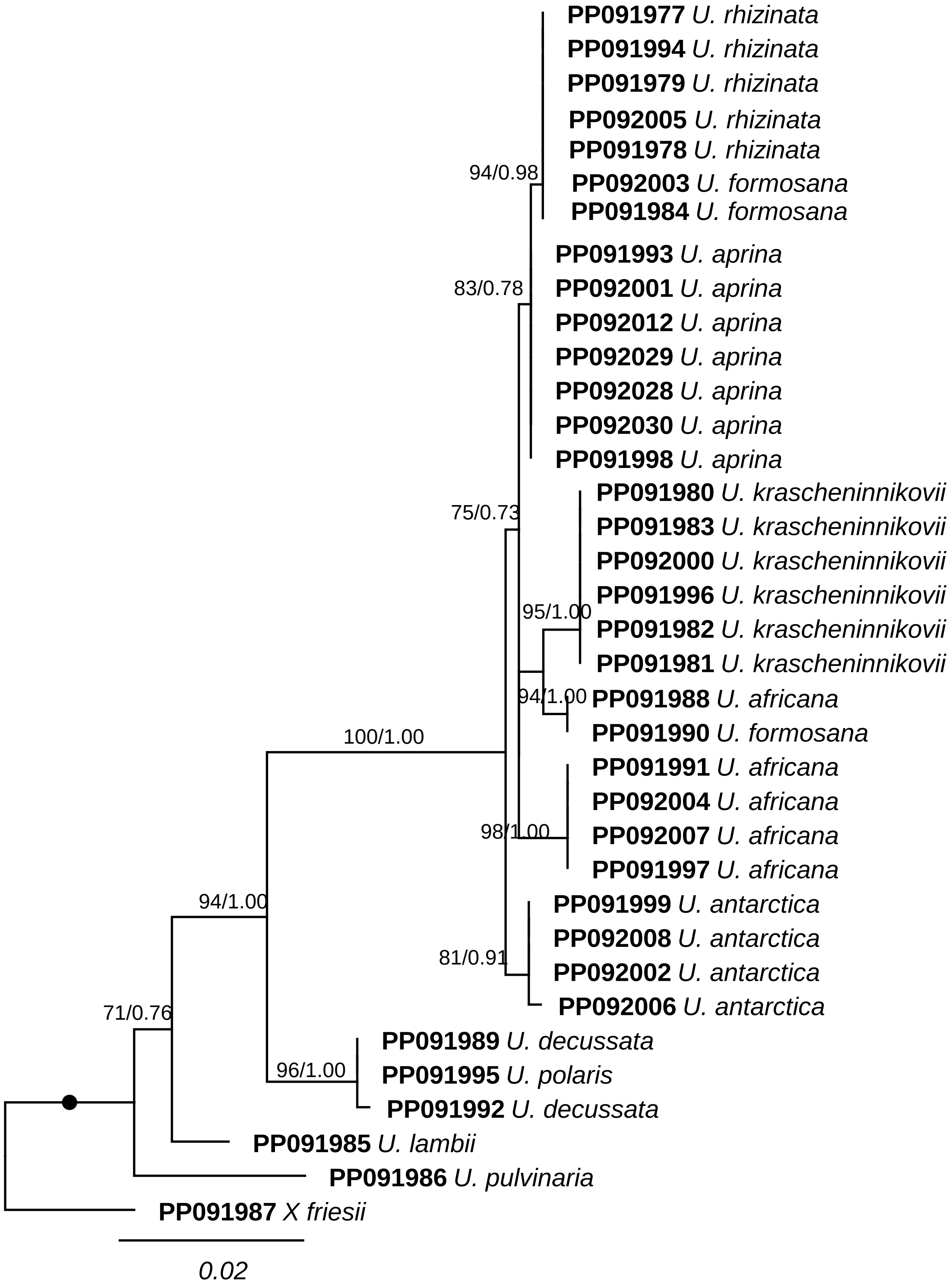

Figure S3. Phylogeny of *Umbilicaria aprina* group based on Maximum Likelihood analysis with IQ-TREE algorithm using the small subunit of the mitochondrial ribosomal DNA (mtSSU) sequences. The species names and GenBank accession numbers are indicated. The number in each node represents bootstrap support (BS) and posterior probability (PP). Branch lengths represent the estimated number of substitutions per site assuming the respective models of substitution. An exception is the branch with a black dot, which was shortened to reduce the overall figure size.

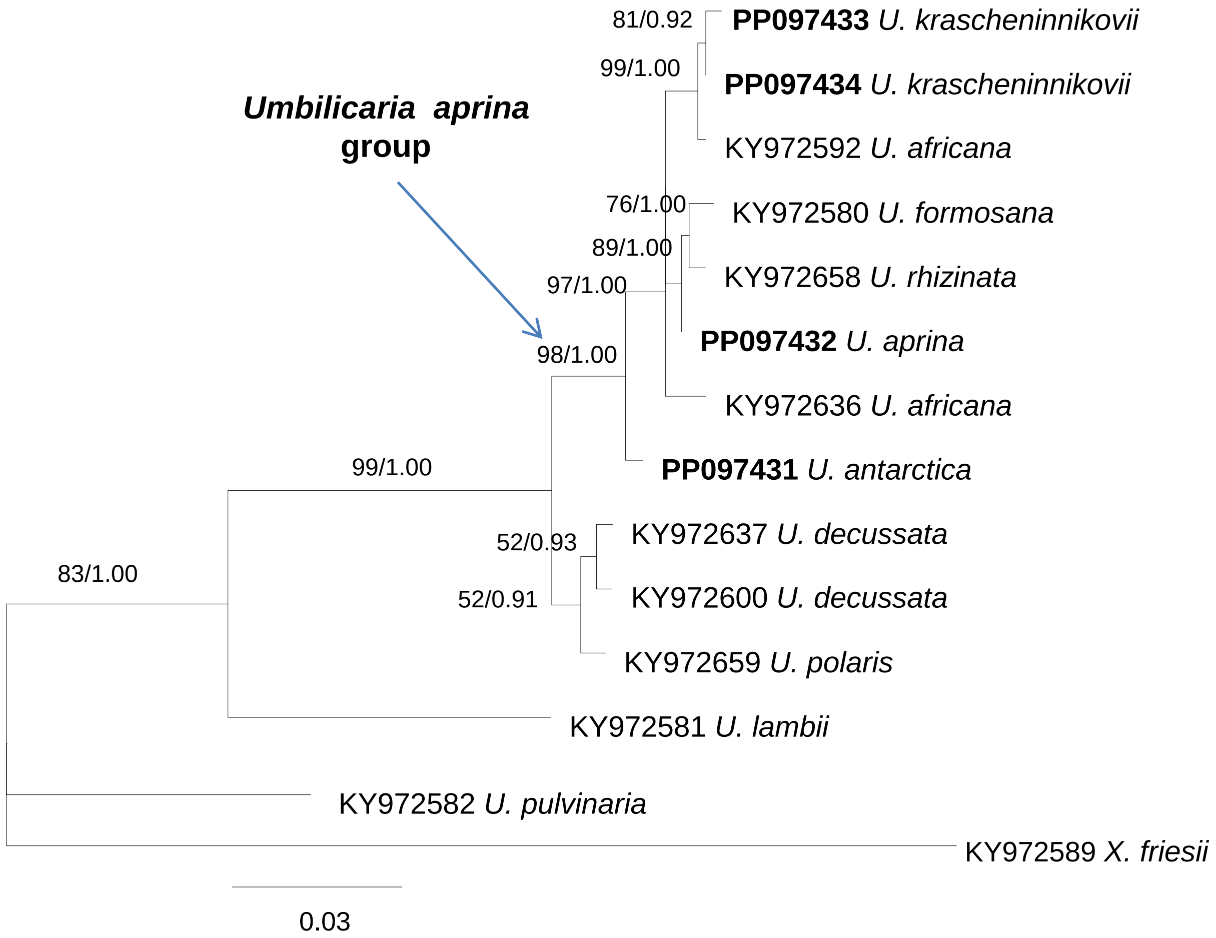

Figure S4. Phylogeny of *Umbilicaria aprina* group based on Maximum Likelihood analysis with IQ-TREE algorithm using the RNA polymerase II (RPB2) gene partial sequences. The species names and GenBank accession numbers are indicated. The number in each node represents bootstrap support (BS) and posterior probability (PP). Branch lengths represent the estimated number of substitutions per site assuming the respective models of substitution.

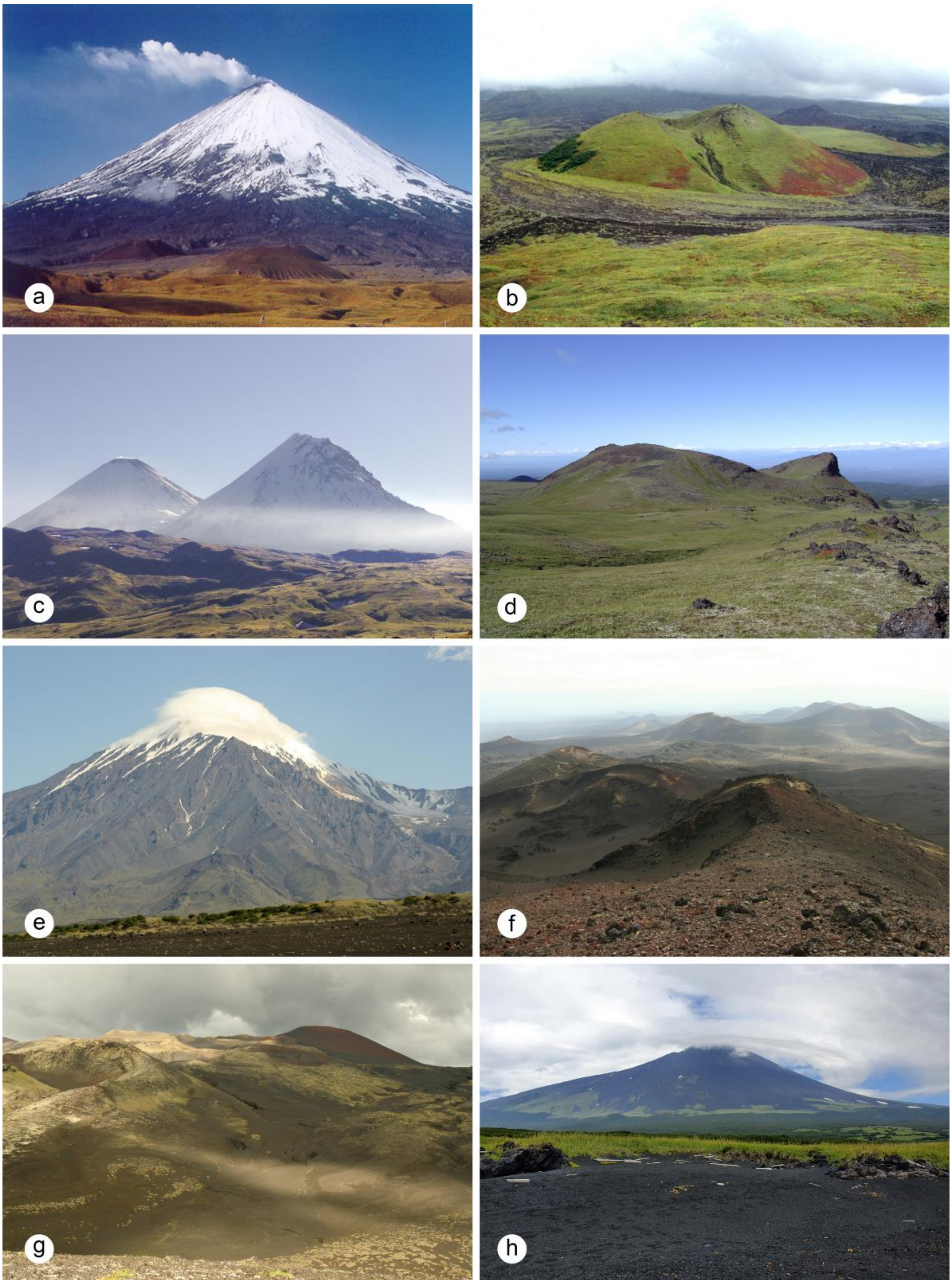

Figure S5. Typical habitats of *Umbilicaria krascheninnikovii* in Kamchatka Peninsula and Kurile Is.: (**a**) SE slope of Kluchevskaya Sopka Volcano; (**b**) Side (parasitic) cone and lava stream Apakhonchich on the SE slope of Kluchevskaya Sopka Volcano; (**c**) Ushkovsky Dale (foreground), volcanoes Kluchevskaya Sopka (left) and Kamen' (right); (**d**) Side cone on the SW slope of Ushkovsky Volcano; (**e**) N slope of Ostry Tolbachik Volcano; (**f, g**) Side cones on the SW slope of Tolbachik Volcano. *Umbilicaria krascheninnikovii* in such localities can be found on lava and volcanic bombs; (**h**) Alaid Volcano in the Atlasova Is.

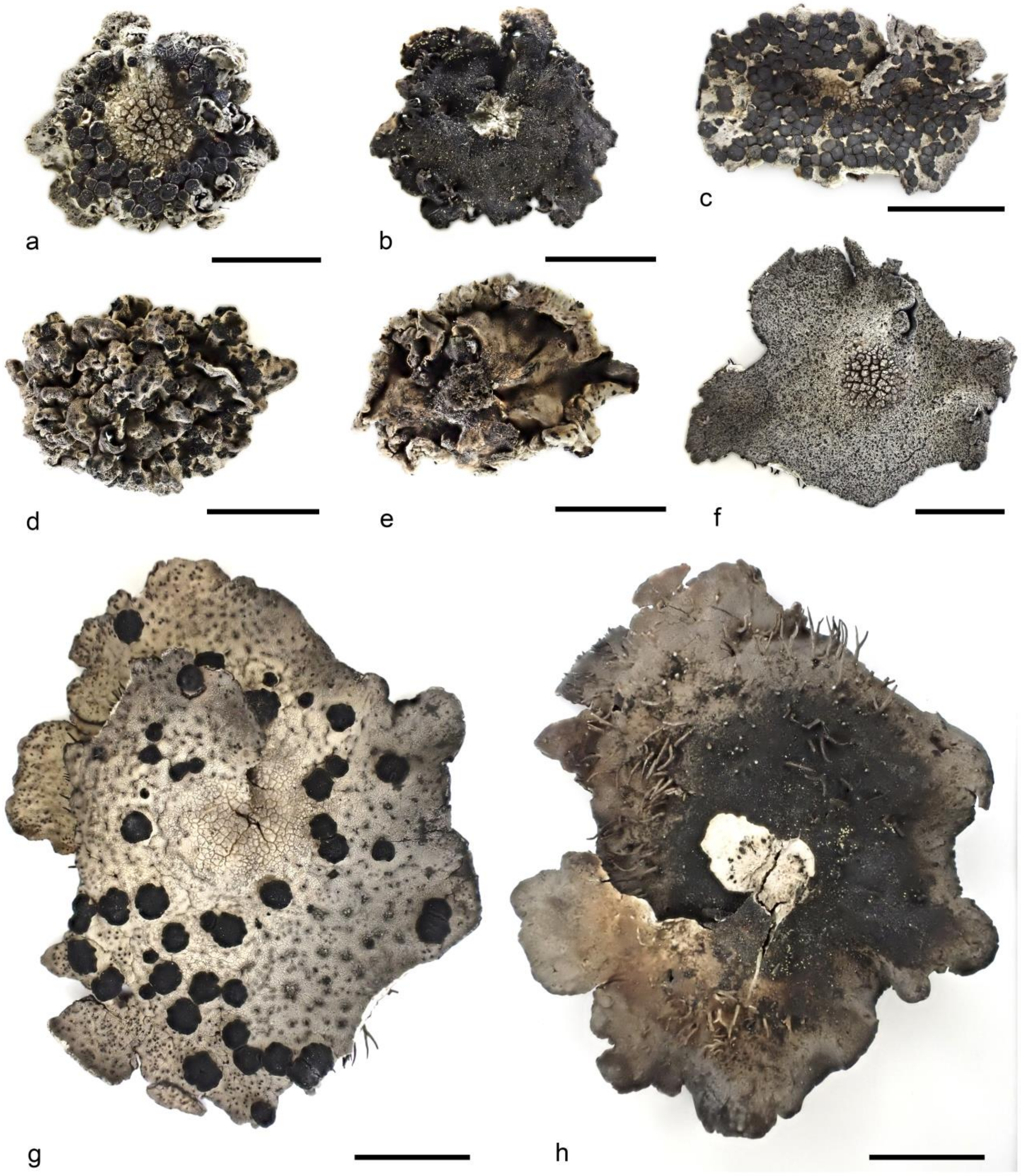

Figure S6. Variability of senescent specimens of *Umbilicaria krascheninnikovii*. A. Upper surface of small-sized senescent thallus with crowded apothecia; B. Entirely black lower surface; C. Upper surface of senescent thallus overgrown with apothecia; D. Irregularly wrinkled upper surface of small-sized senescent thallus with immature apothecia and pycnides; E. Light colored lower surface; F. Rather large senescent thallus without mature apothecia, but with numerous of black dots representing immature apothecia or/and pycnides; G. Uncommonly large senescent thallus with large lobate apothecia; H. Lower surface with scarce rhizinomorphs.

Table S1. Lichen samples used in this study, including voucher information and GenBank accession numbers.

NAME	Isolate	Species name	Collection locality	Collector / Coll. No.	Herbarium / Acc. No.	ITS GB	RPB2	mtLSU	mtSSU
U_africana_1	acpED473	Umbilicaria africana	Ethiopia, Amhara region, Simien Mts. National Park	T. Lutsak 5002	FR-220099	KY947743	KY972592	KY947879	**PP091988**
U_africana_2	agrED352	Umbilicaria africana	Chile, XII Region, Isla Grande de Tierra de Fuego	S. Perez-Ortega 1774	ALTB-L-5466	KY947844		KY947971	**PP091997**
U_africana_3	altED623	Umbilicaria africana	East Antarctica, Enderby Land, Tala Hills	V. E. Myamin	ALTB-L-5503			**PP092024**	**PP092004**
U_africana_4	ED1139	Umbilicaria africana	East Antarctica, Enderby Land, Tala Hills	V. E. Myamin	ALTB	**PP084684**			**PP092007**
U_africana_5	agrED173	Umbilicaria africana (as U. aprina)	East Antarctica, Queen Maud Land, Schirmacher Oasis, Novolazarevskaya station	M. P. Andreev	ALTB-L-158	KY947808	KY972636	KY947931	**PP091991**
U_antarctica_1	agrED367	Umbilicaria antarctica	Antarctic, South Shetland Islands, King George Island, Barton Peninsula	M. P. Andreev 39828	ALTB	KY947849	**PP097431**	KY947978	**PP091999**
U_antarctica_2	altED621	Umbilicaria antarctica	West Antarctica, Galindez Is.	M. P. Andreev	ALTB			**PP092022**	**PP092002**
U_antarctica_3	ED1137	Umbilicaria antarctica	West Antarctica, Galindez Is.	A. Utevskiy	ALTB-L-6769	**PP084682**		**PP092025**	**PP092006**
U_antarctica_4	ED1138	Umbilicaria antarctica	Antarctica, South Shetland Islands, King George Island, Filds Peninsula	M. P. Andreev	ALTB-L-6761	**PP084683**			

U_antarctica_5	ED1140	Umbilicaria antarctica	West Antarctica, Galindez Is.	A. Utevskiy	ALTB-L-6769	**PP084685**		**PP092026**	**PP092008**
U_aprina_2	agrED182	Umbilicaria aprina	Russia, Republic of Buryatia, Khamar-Daban Range, Baikalsky Reserve	G. P. Urbanavichus	ALTB	KY947810		KY947933	**PP091993**
U_aprina_3	agrED360	Umbilicaria aprina	Norway, Svalbard, Longyarbyen	S. Perez-Ortega & S. Domaschke	ALTB-L-5479	KY948014		KY947976	**PP091998**
U_aprina_4	agrED417	Umbilicaria aprina	Norway, Svalbard, Longyarbyen	S. Perez-Ortega & S. Domaschke	ALTB-L-5479	KY947859	**PP097432**	KY947989	**PP092001**
U_aprina_5	ED1387	Umbilicaria aprina	Franz Josef Land, Alexandra Land, Severnaya Bay	S. V. Chesnokov	ALTB-L-7730	OR916138			
U_aprina_6	ED1725	Umbilicaria aprina	Russia, Krasnoyarsk Territory, Sverdrup Is.	L. A. Konoreva	ALTB-L-7371	OR916139		**PP092028**	**PP092009**
U_aprina_7	ED1738	Umbilicaria aprina	Russia, Krasnoyarsk Territory, Sverdrup Is.	L. A. Konoreva	ALTB-L-7372	**PP084686**		**PP092029**	**PP092010**
U_aprina_8	ED1740	Umbilicaria aprina	Russia, Krasnoyarsk Territory, Sverdrup Is.	S. V. Chesnokov	ALTB-L-7373	**PP084687**		**PP092030**	**PP092011**
U_aprina_9	ED1754	Umbilicaria aprina	Russia, Krasnoyarsk Territory, Sverdrup Is.	S. V. Chesnokov	ALTB-L-7370	**PP084688**			**PP092012**
U_aprina_10	ED1792	Umbilicaria aprina	Russia, Republic of Buryatia, Urgedeevsky Range	T. M. Kharpukhaeva	UUH, ALTB-L6594	OR916140			
U_formosana_1	acpED016	Umbilicaria formosana	Russia, Primorye Territory, Snezhnaya Mt.	I. S. Zhdanov	ALTB-L-5494	KY947733	KY972580	KY947865	**PP091984**
U_formosana_2	agrED165	Umbilicaria formosana	China, Yunnan, San Jiang	A. Aptroot 55680	ALTB-5482 ex ABL	KY947806		KY947929	**PP091990**
U_formosana_3	altED622	Umbilicaria formosana	Russia, Primorye Territory, Oblachnaya Mt.	E. A. Davydov 16626	ALTB	**PP084681**		**PP092023**	**PP092003**

U_krascheninnikovii_1	agrED040	Umbilicaria krascheninnikovii	Russia, Kamchatka Peninsula, Kluchevskaya Sopka Volcano	D. E. Himelbrant & E. S. Kuznetsova K-51	ALTB-5488 ex LECB	KY947752		KY947896	
U_krascheninnikovii_2	agrED304	Umbilicaria krascheninnikovii	Russia, Kamchatka Peninsula, Tolbachik Volcano	D. E. Himelbrant & I. S. Stepanchikova K-08-46/47	LE L-7461	**PP084680**	**PP097434**	KY947957	**PP091996**
U_krascheninnikovii_3	agrED414	Umbilicaria krascheninnikovii	Russia, Kamchatka Peninsula, Tolbachik Volcano	D. E. Himelbrant & I. S. Stepanchikova K-226-06	H	KY947857		KY947988	**PP092000**
U_krascheninnikovii_4	acpED007	Umbilicaria krascheninnikovii	Russia, Kamchatka Peninsula, Tolbachik Volcano	D. E. Himelbrant & I. S. Stepanchikova K-08-46	LE L-7461	**PP084672**		**PP092017**	**PP091980**
U_krascheninnikovii_5	acpED008	Umbilicaria krascheninnikovii	Russia, Kamchatka Peninsula, Ushkovsky Volcano	D. E. Himelbrant & E. S. Kuznetsova K-67-04	LE L-7469	**PP084673**		**PP092018**	**PP091981**
U_krascheninnikovii_6	acpED009	Umbilicaria krascheninnikovii	Russia, Kamchatka Peninsula, Tolbachik Volcano	D. E. Himelbrant & I. S. Stepanchikova s. n.	LE L-7501	**PP084674**			
U_krascheninnikovii_7	acpED010	Umbilicaria krascheninnikovii	Russia, Kamchatka Peninsula, Tolbachik Volcano	D. E. Himelbrant & I. S. Stepanchikova K-Alaid-07	LE L-7499	**PP084675**	**PP097433**	**PP092019**	**PP091982**
U_krascheninnikovii_8	acpED012	Umbilicaria krascheninnikovii	Russia, Kamchatka Peninsula, Kluchevskaya Sopka Volcano	D. E. Himelbrant & E. S. Kuznetsova K-64	ALTB, LE L-7498	**PP084676**		**PP092020**	
U_krascheninnikovii_9	acpED013	Umbilicaria krascheninnikovii	Russia, Kamchatka Peninsula, Kluchevskaya Sopka Volcano	D. E. Himelbrant & E. S. Kuznetsova K-51	LE L-7482	**PP084677**		**PP092021**	**PP091983**

U_krascheninnikovii_10	acpED014	Umbilicaria krascheninnikovii	Russia, Kamchatka Peninsula, Kluchevskaya Sopka Volcano	D. E. Himelbrant & E. S. Kuznetsova K-60	ALTB, LE L-7496	**PP084678**			
U_krascheninnikovii_11	acpED015	Umbilicaria krascheninnikovii	Russia, Kamchatka Peninsula, Kluchevskaya Sopka Volcano	D. E. Himelbrant & E. S. Kuznetsova Pesch-Apakh-02	ALTB, LE L-7463	**PP084679**			
U_rhizinata_1	acpED001	Umbilicaria rhizinata	Russia, Republic of Altai, Altai Mts., Katunsky Range	E. A. Davydov 7446	ALTB	OR916141		**PP092013**	**PP091977**
U_rhizinata_2	acpED002	Umbilicaria rhizinata	Russia, Republic of Altai, Altai Mts., Katunsky Range	E. A. Davydov 7421	ALTB	OR916142		**PP092014**	**PP091978**
U_rhizinata_3	acpED003	Umbilicaria rhizinata	Russia, Republic of Altai, Altai Mts., Katunsky Range	E. A. Davydov 6879	ALTB	**PP084670**		**PP092015**	**PP091979**
U_rhizinata_4	acpED004	Umbilicaria rhizinata	Russia, Republic of Altai, Altai Mts., Katunsky Range	E. A. Davydov 7447	ALTB	**PP084671**		**PP092016**	
U_rhizinata_5	acpED006	Umbilicaria rhizinata	Russia, Kamchatka Peninsula, Petropavlovsk-Kamchatsky, Mishennaya Sopka Hill	D. E. Himelbrant & E. S. Kuznetsova K-1	LE L-7465	OR916143			
U_rhizinata_6	agrED295	Umbilicaria rhizinata	Russia, Altai Territory, Altai Mts., Tigireksky Range	E. A. Davydov 7258	ALTB	KY948011	KY972658	KY947954	**PP091994**
U_rhizinata_7	altbED287	Umbilicaria rhizinata	Russia, Altai Territory, Altai Mts., Tigireksky Range	E. A. Davydov 7562	ALTB	OR916144			

U_rhizinata_8	altED638	Umbilicaria rhizinata	USA, Alaska, Gates of the Arctic Park and Reserve	P. Nelson 12-1184	ALTB				**PP092005**
U_rhizinata_9	ED1290	Umbilicaria rhizinata	Russia, Krasnoyarsk Territory, Noril'sk	A.A. Ul'yanovskiy	ALTB	OR916145		**PP092027**	
U_rhizinata_10	ED1789	Umbilicaria rhizinata	Russia, Republic of Buryatia, Nam-Tzagan-Khutliyn-Nuruu Range	T. M. Kharpukhaeva	UUH L01925	OR916146		**PP092031**	
U_rhizinata_11	ED2263	Umbilicaria rhizinata	Russia, Magadan Region, Malyk Lake	E. V. Zheludeva SU4883	MAG	OR916147			
U_rhizinata_12	ED2264	Umbilicaria rhizinata	Russia, Magadan Region, Urultun Lake	E. V. Zheludeva SU4884	MAG	OR916148			
U_lambii	acpED017	Umbilicaria lambii	Canada, British Columbia, Trophy Mt.	M.P Zhurbenko 020702-2	ALTB-L193	KY947734	KY972581	KY947866	**PP091985**
U_pulvinaria	acpED018	Umbilicaria pulvinaria	Russia, Sakhalin Is, Schmidt Peninsula, Elizabeth Cape	S. I. Tchabanenko	ALTB	KY947735	KY972582	KY947867	**PP091986**
U_decussata_1	agrED022	Umbilicaria decussata	Kazakhstan, Altai Mts., Saur Range	D. A. German	ALTB-L153	KY948001	KY972600	KY947891	**PP091989**
U_decussata_2	agrED174	Umbilicaria decussata	Antarctica, Hasuell Is.	M. P. Andreev 041601	ALTB-L157	KY947809	KY972637	KY947932	**PP091992**
U_polaris	agrED296	Umbilicaria polaris	Russia, Altai Territory, Altai Mts., Tigireksky Range	E. A. Davydov 7251	ALTB	KY947830	KY972659	KY947955	**PP091995**
Xylopsora_friesii	acpED467	Xylopsora friesii	Russia, Murmansk Region, Pasvik Reserve	G. P. Urbanavichus	ALTB-L5425	KY947740	KY972589	KY947876	**PP091987**

Table S2. Examined specimens of *Umbilicaria kraschenninikovii* .

Collection region	Locality	Coordinates	Altitude	Biotope	Date	Collector / Coll. No.	Herbarium /No.	Isolate
Russia, Kamchatka Peninsula	Central Kamchatka, Ust'-Kamchatsk District, c. 30 km S of Kluchi, E slope of volcano Kluchevskaya Sopka, lava stream Apakhonchich	56˚01'42''N, 160˚45'02''E	1580 m a. s. l.	on stones	26.08.2002	D. E. Himelbrant & E. S. Kuznetsova K-51	ALTB-5488 ex LECB	agrED040
Russia, Kamchatka Peninsula	Central Kamchatka, Mil'kovo District, Kamchatka River basin, SW slope of Tolbachik volcano, c. 40 km SE of Kozyrevsk	55˚46'35''N, 160˚18'42''E	1630 m a. s. l.	on lava stream	16.08.2008	D. E. Himelbrant & I. S. Stepanchikova K-08-46/47	LE L-7461	agrED304
Russia, Kamchatka Peninsula	Central Kamchatka, Mil'kovo District, Kamchatka River basin, SW slope of Tolbachik volcano, ca. 40 km SE of Kozyrevsk. Lava stream of 1941	55˚46'55''N, 160˚17'18''E	1429 m a. s. l.	stony mossy tundra with dwarf shrubs on the slope	11.08.2006	D. E. Himelbrant & I. S. Stepanchikova K-226-06	H	agrED414
Russia, Kamchatka Peninsula	Central Kamchatka, Mil'kovo District, Kamchatka River basin, SW slope of Tolbachik volcano, c. 40 km SE of Kozyrevsk	55˚46'35''N, 160˚18'42''E	1630 m a. s. l.	on lava stream	16.08.2008	D. E. Himelbrant & I. S. Stepanchikova K-08-46	LE L-7461	acpED007
Russia, Kamchatka Peninsula	Central Kamchatka, Ust'-Kamchatsk District, c. 24 km SE of Kozyrevsk, Kamchatka River basin, SW slope of Ushkovsky volcano	55˚57'51.3''N, 160˚14'28.2''E	1080 m a. s. l.	on lava stones	14.08.2004	D. E. Himelbrant & E. S. Kuznetsova K-67-04	LE L-7469	acpED008
Russia, Kamchatka Peninsula	Central Kamchatka, Mil'kovo District, Southern slope of Tolbachik Volcano	55˚44'N, 160˚11'E	c. 700 m a. s. l.	on lava outcrops	10.08.2006	D. E. Himelbrant & I. S. Stepanchikova s. n.	LE L-7501	acpED009

Russia, Kamchatka Peninsula	Central Kamchatka, Mil’kovo District, c. 44 km SE of Kozyrevsk, Kamchatka River basin, SW slope of Tolbachik volcano, Alaid lava stream	55˚43'N, 160˚13'E	1330 m a. s. l.	on lava stream	29.08.2007	D. E. Himelbrant & I. S. Stepanchikova K-Alaid-07	LE L-7499	acpED010
Russia, Kamchatka Peninsula	Central Kamchatka, Ust’-Kamchatsk District, c. 30 km S of Kluchi, E slope of volcano Kluchevskaya Sopka, lava stream Apakhonchich	55˚59'38"N, 160˚50'29"E	770 m a. s. l.	lava outcrops with seldom Salix shrubs	29.08.2002	D. E. Himelbrant & E. S. Kuznetsova K-64	LE L-7498	acpED012
Russia, Kamchatka Peninsula	Central Kamchatka, Ust’-Kamchatsk District, c. 30 km S of Kluchi, E slope of volcano Kluchevskaya Sopka, lava stream Apakhonchich	56˚01'42"N, 160˚45'02"E	1580 m a. s. l.	on stones	26.08.2002	D. E. Himelbrant & E. S. Kuznetsova K-51	LE L-7482	acpED013
Russia, Kamchatka Peninsula	Central Kamchatka, Ust’-Kamchatsk District, c. 30 km S of Kluchi, E slope of volcano Kluchevskaya Sopka, lava stream Peschernyj	55˚59'N, 160˚47'E	1050 m a. s. l.	on stones	28.08.2002	D. E. Himelbrant & E. S. Kuznetsova K-60	LE L-7496	acpED014
Russia, Kamchatka Peninsula	Central Kamchatka, Ust’-Kamchatsk District, E slope of Kluchevskaya Sopka volcano, c. 30 km S of Kluchi, between lava streams Apakhonchich and Peschernyj	55˚59'N, 160˚49'E	c. 900 m a. s. l.	on lava	28.08.2002	D. E. Himelbrant & E. S. Kuznetsova Pesch-Apakh-02	LE L-7463	acpED015
Russia, Kamchatka Peninsula	SE Kamchatka, Elizovo District, Petropavlovsk-Kamchatsky, E slope of Mishennaya Sopka Hill	53°02'36"N, 158°38'28"E	370 m a. s. l.	lava outcrops	29.07.2002	D. E. Himelbrant & E. S. Kuznetsova K-1	LE L-7466	

Russia, Kamchatka Peninsula	SE Kamchatka, Elizovo District, Kronotsky Nature Reserve, 5 km S of Krasheninnikova Volcano, vicinity of Svetlaya River	54˚30'N, 160˚17'E	c. 400 m a. s. l.	lava stream, on lava	06.08.1985	A. Mikulin 1-653-d	LE L-5778	
Russia, Kamchatka Peninsula	Central Kamchatka, Ust'-Kamchatsk District, c. 30 km S of Kluchi, E slope of volcano Kluchevskaya Sopka, lava stream Apakhonchich	56˚01'20.4''N, 160˚45'51.1''E	1420 m a. s. l.	on stones	26.08.2002	D. E. Himelbrant & E. S. Kuznetsova K-53	LE L-6887	
Russia, Kamchatka Peninsula	Central Kamchatka, Ust'-Kamchatsk District, c. 18 km S of Kluchi, NE slope of the volcano Kluchevskaya Sopka, near crater and volcanic station Podkova	56˚07'54.2''N, 160˚47'00.8''E	1040 m a. s. l.	on stones	31.08.2002	D. E. Himelbrant & E. S. Kuznetsova K-70	LE L-7467	
Russia, Kamchatka Peninsula	Central Kamchatka, Ust'-Kamchatsk District, c. 40 km SE of Kozyrevsk, Kamchatka River basin, SW slope of Ushkovsky volcano	55˚55'49.4''N, 160˚19'37.8''E	1030 m a. s. l.	Larix cajanderi sparse forest, on stones	21.08.2005	D. E. Himelbrant & E. S. Kuznetsova K-189-05	LE L-7472	
Russia, Kamchatka Peninsula	Central Kamchatka, Mil'kovo District, S slope of Tolbachik Volcano, c. 44 km SE of Kozyrevsk, Alaid tephra field	55˚42'32''N, 160˚13'24''E	860 m a. s. l.	community on lava sprinkled with scoria, on lava	22.08.2007	D. E. Himelbrant & I. S. Stepanchikova K-36-07	LE L-7473	
Russia, Kamchatka Peninsula	Central Kamchatka, Mil'kovo District, c. 44 km SE of Kozyrevsk, Kamchatka River basin, SW slope of Tolbachik volcano, Alaid lava stream	55˚43'N, 160˚13'E	c. 1300 m a. s. l.	on stones	29.08.2007	D. E. Himelbrant & I. S. Stepanchikova 1107-07	LE L-7477	

Russia, Kamchatka Peninsula	Central Kamchatka, Mil'kovo District, c. 44 km SE of Kozyrevsk, Kamchatka River basin, SW slope of Tolbachik volcano, Alaid lava stream	55˚43'N, 160˚13'E	c. 1300 m a. s. l.	on stones	29.08.2007	D. E. Himelbrant & I. S. Stepanchikova 1107-07	LE L-7478	
Russia, Kamchatka Peninsula	Central Kamchatka, Ust'-Kamchatsk District, c. 45 km NE of Kluchi, W slope of volcano Shiveluch	56˚35'16''N, 161˚12'59''E	1110 m a. s. l.	lichen-herb tundra, on stones	24.08.2002	D. E. Himelbrant & E. S. Kuznetsova K-50	LE L-7479	
Russia, Kamchatka Peninsula	Central Kamchatka, Ust'-Kamchatsk District, c. 45 km NE of Kluchi, W slope of volcano Shiveluch	56˚35'16''N, 161˚12'59''E	1110 m a. s. l.	lichen-herb tundra, on stones	24.08.2002	D. E. Himelbrant & E. S. Kuznetsova K-50	LE L-7480	
Russia, Kamchatka Peninsula	Central Kamchatka, Ust'-Kamchatsk District, c. 30 km S of Kluchi, E slope of volcano Kluchevskaya Sopka, lava stream Apakhonchich	56˚01'20.4''N, 160˚45'51.1''E	1420 m a. s. l.	on stones	26.08.2002	D. E. Himelbrant & E. S. Kuznetsova K-53	LE L-7485	
Russia, Kamchatka Peninsula	Central Kamchatka, Ust'-Kamchatsk District, c. 30 km S of Kluchi, E slope of volcano Kluchevskaya Sopka, lava stream Apakhonchich	56˚01'20.4''N, 160˚45'51.1''E	1420 m a. s. l.	on stones	26.08.2002	D. E. Himelbrant & E. S. Kuznetsova K-53	LE L-7486	
Russia, Kamchatka Peninsula	Central Kamchatka, Ust'-Kamchatsk District, c. 30 km S of Kluchi, E slope of volcano Kluchevskaya Sopka, lava stream Apakhonchich	56˚01'08''N, 160˚46'24.3''E	1300 m a. s. l.	on stones	26.08.2002	D. E. Himelbrant & E. S. Kuznetsova K-54	LE L-7489	
Russia, Kamchatka Peninsula	Central Kamchatka, Ust'-Kamchatsk District, c. 30 km S of Kluchi, E slope of volcano Kluchevskaya Sopka, lava stream Apakhonchich	56˚00'52.4''N, 160˚47'24.1''E	1140 m a. s. l.	on stones	26.08.2002	D. E. Himelbrant & E. S. Kuznetsova K-56	LE L-7493	

Russia, Kamchatka Peninsula	Central Kamchatka, Ust'-Kamchatsk District, c. 30 km S of Kluchi, E slope of volcano Kluchevskaya Sopka, lava stream Apakhonchich	56˚00'52.4"N, 160˚47'24.1"E	1140 m a. s. l.	on stones	26.08.2002	D. E. Himelbrant & E. S. Kuznetsova K-56	LE L-7494	
Russia, Kamchatka Peninsula	Central Kamchatka, Ust'-Kamchatsk District, c. 30 km S of Kluchi, E slope of volcano Kluchevskaya Sopka, lava stream Bilukaj	56˚03'23.0"N, 160˚50'53.5"E	830 m a. s. l.	on stones	27.08.2002	D. E. Himelbrant & E. S. Kuznetsova K-59	LE L-7495	
Russia, Kurile Is.	Araito Is. [=Atlasova Is.]: on route from Minami-ura to Ichino-watashi				15.07.1931	Y. Okada	TNS	
Japan. Honshu Is.	Shizuoka-ken, Sunto-gun, Oyama-cho, Mt. Fuji		ca. 3700 m a. s. l.	on rock	10.09.2002	K. Takahashi 1808	HIRO	
